# Pan-Cancer Landscape of Magnesium Homeostasis: Bulk Omics Research and Single-Cell Sequencing Validation

**DOI:** 10.1186/s12575-025-00294-1

**Published:** 2025-10-31

**Authors:** Yan Qin, Zhuo-er Yuan, De Yin, Sheng-yue Zhang, Yu-cao Sun, Wei Li

**Affiliations:** 1 Department of Health Management, Zhuang Autonomous Region & Research Center of Health Management, The People’s Hospital of Guangxi, Guangxi Academy of Medical Sciences, Nanning, 530021 Guangxi People’s Republic of China; 2 Guangxi Medical University Cancer Hospital, Nanning, 530021 Guangxi People’s Republic of China

**Keywords:** Magnesium homeostasis, Tumor immunity, Tumor microenvironment, Single-cell sequencing validation

## Abstract

Introduction: Magnesium homeostasis is critical for cellular growth and metabolism, yet its pan-cancer implications remain poorly characterized. This study aims to comprehensively analyze magnesium homeostasis across 33 cancer types, exploring its role in tumorigenesis, immune regulation, and therapeutic potential. Key magnesium homeostasis-related genes (e.g., ANK3, CNNM2) were significantly downregulated in most tumors, correlating with improved prognosis. Magnesium homeostasis scores (MHS) were reduced in cancers and linked to lower tumor mutational burden (TMB), microsatellite instability (MSI), immune dysfunction, and checkpoint gene expression. Single-cell sequencing revealed elevated MHS in CD8 + T cells, suggesting immune modulation roles. Conclusion: These findings highlight magnesium homeostasis as a regulator of tumor progression and immunity, with MHS serving as a prognostic biomarker. Targeting magnesium pathways may offer novel therapeutic strategies, warranting further clinical validation to advance personalized cancer therapies.

**Supplementary Information:**

The online version contains supplementary material available at 10.1186/s12575-025-00294-1.

## Introduction

Cancer is the second leading cause of mortality worldwide, with its incidence and mortality increasing every year [1]. Hence, there is an urgent need to explore and study the mechanism of tumor genesis and development. Recently, there has been an increasing interest in the relationship between metal ion metabolism and tumor development. For example, ferroptosis is an iron-dependent form of regulated cell death, and several studies have explored the design and development of ferroptosis-based anti-cancer drugs as a novel approach to tumor treatment [2]. Further, cuprotosis is another hot topic in anti-tumor research. It occurs through the direct binding of copper ions to lipid acylated components of the tricarboxylic acid cycle in mitochondrial respiration, leading to lipid acylated protein aggregation and subsequent downregulation of iron-sulfur cluster proteins, resulting in proteotoxic stress and ultimately cell death [3]. Many studies, both in preclinical and clinical settings, have investigated the interaction of magnesium with key mediators of the physiological stress response and have demonstrated that magnesium plays an inhibitory key role in the regulation and neurotransmission of the normal stress response [4]. However, the association between magnesium homeostasis and tumor development and progression is still unknown.

Magnesium is one of the major cations in biological systems. As a cofactor of ATP, Mg^2+^ is involved in more than 600 enzymatic reactions and is pivotal for numerous biological mechanisms, including DNA transcription, protein synthesis, and energy metabolism [5]. It also regulates immune function, acting on the cells of the innate and adaptive immune system. Magnesium deficiency promotes phagocytosis, enhances granulocyte set oxidative burst, activates endothelial cells and increases the level of cytokines, thus promoting inflammation [6]. In addition, most ATP in the cell is Mg2 + complexed and most ATPases use Mg-ATP [7]. Thus, alterations in Mg2 + could have significant consequences for energy producing and utilizing enzymes. Although the large number of enzymes and transporters modulated by Mg2 + presents an opportunity for the cell to coordinately alter cell function [8]. Thus, it is important to maintain and regulate magnesium levels according to the metabolic state of the cell [9]. Disturbances in magnesium homeostasis lead to magnesium deficiency which is common in various diseases, including diabetes, cardiovascular diseases, chronic fatigue, alcoholism, and psychiatric and neurological disorders. Severe hypomagnesemia may lead to disorders of the neuromuscular and cardiovascular systems [10]. Reduced intracellular Mg^2+^ levels also inhibit cell cycle progression, and therefore Mg^2+^ is associated with impaired cell growth [11].Further, in magnesium-deficient mice, low magnesium both limits and promotes tumorigenesis, as tumor growth inhibition at their primary sites were observed along with increased metastatic colonization [12].On the one hand, magnesium deficiency impairs the function of immune cells, reducing their ability to recognize and destroy tumor cells. On the other hand, disruption of magnesium homeostasis may also affect the tumor microenvironment, such as by regulating the production and release of inflammatory cytokines, which in turn affect the infiltration and function of immune cells.

First, magnesium is essential for DNA transcription and protein synthesis, a fundamental process of cell division and growth. Magnesium deficiency can lead to errors in these processes, potentially leading to mutations and abnormal cell proliferation. This can create an environment conducive to the development of tumors.

Secondly, magnesium plays a regulatory role in immune function, affecting innate and adaptive immune responses. It has been shown to regulate phagocytosis, granulocyte oxidative breakdown, endothelial cell activation, and cytokine production. By affecting these immune processes, magnesium can influence the inflammatory response, which is often uncontrolled in cancer. Magnesium deficiency may cause an immune system imbalance, allowing cancer cells to evade immune surveillance and proliferate.

In this study, we conducted comprehensive analyses of differential gene expression, protein interaction, pathways, and prognosis of magnesium homeostasis-related genes in the pan-cancer environment. We identified 13 genes related to magnesium homeostasis and investigated the related functions of magnesium homeostasis at the single-cell level for elucidating the relationship between magnesium homeostasis and pan-cancer. We further examined the anti-tumor effects of surface magnesium homeostasis.

## Methods

### Data Analysis and Processing

Single cell sequencing data was derived from GEO database (05/2022) [13]. The sequencing and initial processing of the data were conducted as follows. The analyzed samples were initially sequenced using Hiseq X10 [14](Illumina, San Diego, CA, USA) standard parameters. Subsequently, the sequencing file (BCL) was converted to FASTQ file form on Cell Ranger (Version 3.0.2) R (Version 3.5.2) [15]. Finally, the samples were analyzed for QC and secondary analysis using the “Seurat” R package (Version 3.1.1).

### Source and Preprocessing of Data

Clinical data and gene expression data used in this experiment were obtained from the TCGA database (04/2022) [16] (https://portal.gdc.cancer.gov/) and The Pan-Cancer Atlas Center at the University of California, Santa Cruz (UCSC [16]) database (04/2022). The analysis included samples from 33 solid cancers (Supplementary Table 1), namely adrenocortical carcinoma (ACC), bladder urothelial carcinoma (BLCA), lymphoid neoplasm diffuse large B-cell lymphoma (DLBC), esophageal carcinoma (ESCA), glioblastoma multiforme (GBM), head and neck squamous cell carcinoma (HNSC) breast invasive carcinoma (BRCA), cervical squamous cell carcinoma and endocervical adenocarcinoma (CESC), cholangiocarcinoma (CHOL), colon adenocarcinoma (COAD), kidney chromophobe (KICH), kidney renal clear cell carcinoma (KIRC), kidney renal papillary cell carcinoma (KIRP), acute myeloid leukemia (LAML), brain low-grade glioma (LGG), liver hepatocellular carcinoma (LIHC), lung adenocarcinoma (LUAD), lung squamous cell carcinoma (LUSC), mesothelioma (MESO), ovarian serous cystadenocarcinoma (OV), pancreatic adenocarcinoma (PAAD), pheochromocytoma and paraganglioma (PCPG), prostate adenocarcinoma (PRAD), rectum adenocarcinoma (READ), sarcoma (SARC), skin cutaneous melanoma (SKCM), stomach adenocarcinoma (STAD), testicular germ cell tumors (TGCT), thyroid carcinoma (THCA), thymoma (THYM), uterine corpus endometrial carcinoma (UCEC), uterine carcinosarcoma (UCS), and uveal melanoma (UVM).

### Differential Gene Expression Analysis

Through in-depth analysis of the expression levels of different genes, the expression differences of magnesium homeostasis related genes in tumor tissues and normal tissues were revealed. The tumor specimens were divided into two groups according to the best cut point, and the survival time and survival status of the two groups were fitted by “survival” R package.

### Tumor Immune Dysfunction and Exclusion Analysis

The Tumor immune dysfunction and exclusion (TIDE) algorithm predicts potential ICB responses. TIDE uses a suite of gene expression markers to assess two different mechanisms of tumor immune evasion, including tumor-infiltrating cytotoxic T lymphocyte (CTL) [17]dysfunction and CTL rejection by immunosuppressive factors. The probability of tumor immune escape was higher in patients with a high TIDE score than those with a low TIDE score. Therefore, patients with a high TIDE score showed a lower rate of ICB response.

### Estimation of Magnesium Homeostasis Score

In order to quantify the expression level of magnesium homeostasis related genes, the magnesium homeostasis related gene set downloaded from GSEA database (04/2022) (MAGNESIUM_ION_HOMEOSTASIS) [18] was calculated by single-sample gene set enrichment analysis (ssGSEA) to explore the magnesium homeostasis score. The ssGSEA analysis was performed using the packages “GSEABase [19],"“limma [20],"and “GSVA [21]” in R, using default parameters. The standardized enrichment score was used as the magnesium homeostasis score.

### Construction of Magnesium Homeostasis Regulatory Network and Protein–Protein Interaction (PPI) Analysis

Magnesium homeostasis related genes were imported into STRING database (05/2025) (https://string-db.org/) for protein-protein interaction (PPI) analysis. Then download it. txt file was copied to excel for annotation, and Cytoscape software was imported to generate PPI network map of core genes. The network topology was analyzed by Cytoscape network analysis function, and genes with a defined degree greater than 30 were considered as hub genes.

### Paraffin-Embedded Tissue Collection

The selected pairs of normal and para-cancer tissues were obtained from 35 breast cancer patients. All 35 patients had breast cancer and were not treated with chemotherapy or radiotherapy before the relevant cancer tissue was collected. Written informed consent was obtained from all patients. This study was approved by the Ethical Anthropology Committee. All experiments and methods were performed in accordance with the relevant guidelines and regulations.

### Immunohistochemical Staining

 The collected tissues were prepared into tissue microarrays. When dewaxed and rehydrated at 37 °C and incubated in 5% BSA for 30 min, endogenous peroxidase activity was inhibited and blocked. Treated tumour sections were incubated overnight with anti-TRPM7 (XY-Bioscience XY00019) at 4 °C and then washed three times with phosphate buffered saline (PBS) solution. Tumour sections were then incubated in secondary antibodies labeled with horseradish peroxidase at 37 °C for 30 min. After washing in PBS solution for three times, the sections were developed in diaminobenzidine, and microscopic imaging was performed by light mirror.

### Copy-Number Variant (CNV) Analysis

We mainly used CNV data of 32 different tumor types and conducted a series of analyses and studies using GISTICS2.0 for these tumor types (04/2022). CNV can be divided into heterozygous and homozygous types according to type, which also represent different meanings. Specifically, CNVS are found on several chromosomes, one or two, in each cancer. Heterozygous and homozygous CNV plots show the percentage of CNV amplification and deletion for each gene in each cancer. We use the Gisticc processed data to calculate the percentage of CNV subtypes. Finally, correlation analysis was used to detect the correlation between paired mRNA expression and CNV percentage.

### Standard Normal Variate Transform (SNV) Analysis

SNV data from 32 different tumors generated by MAFTools were analyzed (04/2022). Based on these data, Silent, Intron, IGR, 3 UTR, 5 UTR, 3 flanks and 5 flanks were selected to analyze the SNV ratio. The percentage of SNV was then obtained by dividing the number of mutant samples by the number of cancer samples. Finally, SNV data were combined with clinical survival data to predict survival inequality between mutated and non-mutated genes using an R software package.

### Methylation Analysis

Methylation data were analyzed from 14 different tumor and normal tissue samples (04/2022), then combined with mRNA expression and methylation data using TCGA barcodes and Pearson correlation coefficient and T-test to analyze the data. Then, RPPA in TCPA was used to score and analyze the samples to obtain the relative protein levels, which were divided into up-regulation group and down-regulation group, and then the pathway activity score was measured. The miRNA regulatory network was used to analyze the miRNA regulatory data in TCGA of 32 cancer types. TCGA barcoding was used to fuse miRNA expression and gene expression, and Pearson correlation coefficient and T-test were used to detect the association between paired mRNA and miRNA expression.

### Drug Sensitivity Analysis

Data were collected through Cancer Treatment Response Portal (GDSC) (04/2022) to analyze the relationship between drug sensitivity and genes, obtain the AUC value of tumor cell line drugs, and conduct correlation analysis between gene transcription level and Pearson correlation coefficient. Pearson correlation coefficients of drug target pair markers were compared with the same number of correlation pairs generated by random sampling. Genomic drug resistance analysis based on IC50 drug data.

## Results

### Differential Gene Expression Analysis and Prognostic Analysis of Hub Genes Associated with Magnesium Homeostasis

**Fig. 1:**
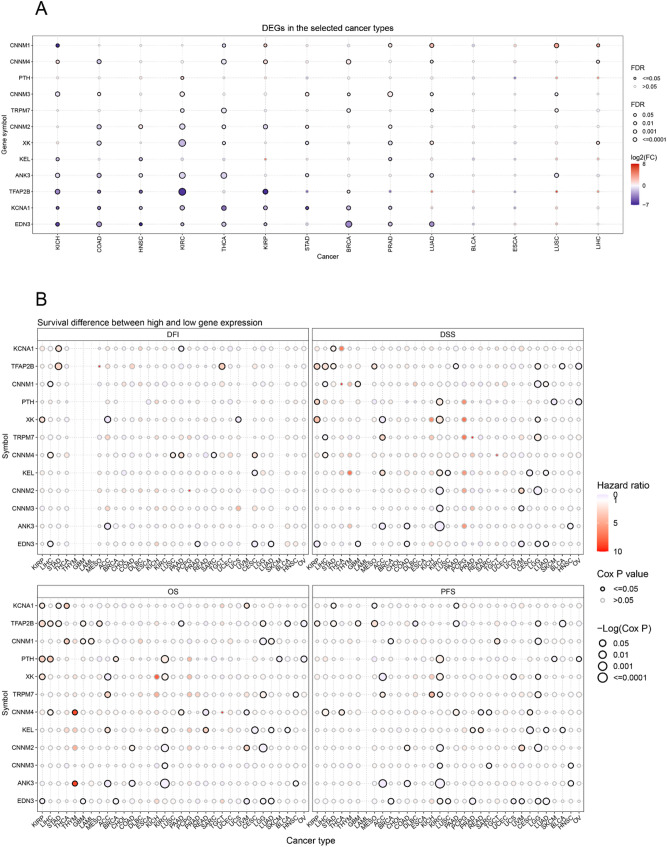
Expression and prognosis of magnesium homeostasis related hub genes. **A** Differences in the expression of key genes related to magnesium homeostasis between tumor and non-cancerous tissues. FDR, false discovery rate. **B** Relationship between magnesium homeostasis related hub genes and disease-free interval (DFI), disease-specific survival (DSS), overall survival (OS) and progression-free survival (PFS)

To gain a comprehensive understanding of the impact of magnesium homeostasis-related genes (MHGs) on the development of various cancers, we performed protein interaction network analysis and analyzed the topology of the network using clinical data from The Cancer Genome Atlas (TCGA) database, and finally screened 13 key differential genes related to magnesium homeostasis (Supplementary Fig. 1). We found significant variability in the expression of almost all genes in cancer and non-cancerous tissues. Most of the genes were down-regulated in tumor tissues, for example, EDN3, KCNA1, TFAP2B, and ANK3 genes were significantly down-regulated in KICH, COAD, HNSC, KIRC, THCA, and KIRP. Meanwhile, CNNM1 gene was downregulated in KICH and THCA but upregulated in LUAD, LUSC and LIHC (Fig. 1A). The results showed that magnesium homeostasis-related genes were negatively associated with tumor progression. Next, based on prognostic analysis of MHGs, we found that these key genes correlated with the prognosis of multiple cancers. In overall survival (OS), some genes can be considered as protective factors. The downregulation of their expression is associated with better prognosis in several cancers, such as XK in ACC, CNNM4 in READ, KEL in CESC, CNNM3 in KIRC, as well as CNNM2 in LGG and KIRC (Fig. 1B).

### CNVs and SNVs of Hub Genes Associated with Magnesium Homeostasis

**Fig. 2:**
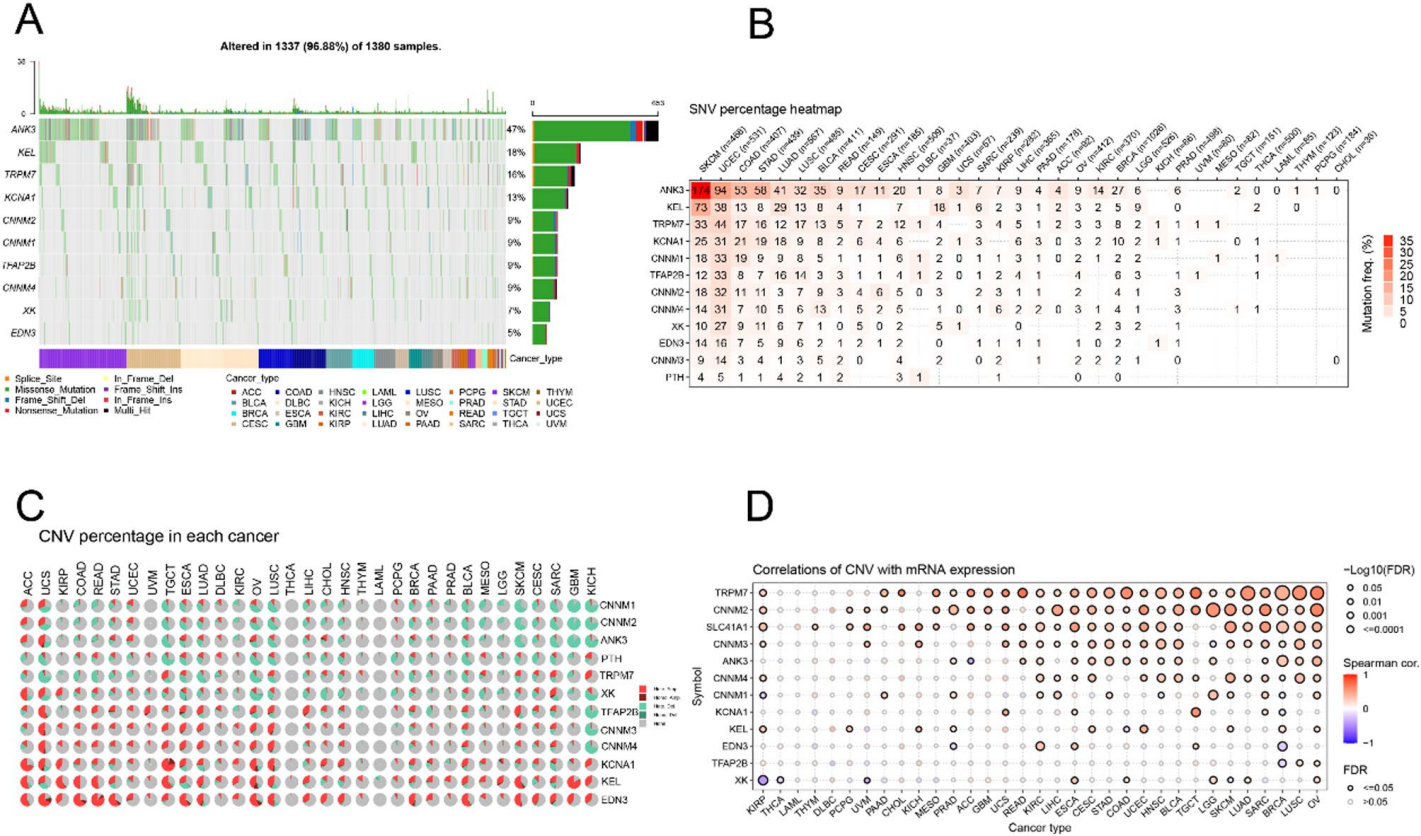
Genomic alterations in magnesium homeostasis-associated hub genes. **A** Genetic mutations in magnesium homeostasis-associated hub genes in pan-cancer. **B** Heatmap showing the Single Nucleotide Varients (SNV) percentage of magnesium homeostasis-associated hub genes in pan-cancer from The Cancer Genome Atlas (TCGA) database. **C** Type of CNV in pan-cancer. The area indicates the CNV percentage. **D** Correlations of CNV with mRNA expression of magnesium homeostasis-associated hub genes. Red indicates a positive correlation, and purple indicates a negative correlation. *FDR* false discovery rate

Based on TCGA, we evaluated single-nucleotide variants (SNVs) and copy number variations (CNVs) of MHGs. We observed that 1337 of 1380 samples (96.88%) showed at least one magnesium homeostasis-related gene mutation. The mutation rates of different MHGs varied widely across cancer tissues, with ANK3 having the highest mutation rate (47%), followed by KEL (18%), TRPM7 (16%), KCNA1 (13%), CNNM2 (9%), CNNM1 (9%), TFAP2B (9%), CNNM4 (9%), XK (7%), and EDN3 (5%). The main SNV types were missense mutations, nonsense mutations, frameshift deletions, splice sites, frameshift insertions, in-frame deletions, and in-frame. In addition, the frequency of SNVs was higher in SKCM, UCEC, LUAD, STAD, COAD, and LUSC than in other cancers (Fig. 2A). According to the proportion of SNVs in MHG, ANK3 was mutated at a high frequency in SKCM, UCEC, COAD, STAD, LUAD, LUSC, and BLCA. Some cancer types, such as SKCM (37.18%), UCEC (17.70%), STAD (13.21%) and COAD (13.02%), had the highest frequency of gene mutations (Fig. 2B). Upon analysis of the proportion of CNVs in pan-cancer, we found that the main types of CNVs in MHGs in TCGA tumors were heterozygous amplifications and heterozygous deletions. KCNA1, KEL and EDN3 mainly showed heterozygous amplifications, including ACC, UCS, KIRP, COAD, READ, OV and LUSC; while CNNM1, CNNM2 and ANK3 mainly showed heterozygous deletions, including SKCM, CESC, SARC, GBM, KICH and TGCT (Fig. 2C). mRNA expression of MHG was positively correlated with CNV in most cancers, such as SLC41A1, CNNM2, and TRPM7 in SKCM, LUAD, SARC, BRCA, LUSC and OV (*P* < 0.05) (Fig. 2D). Thus, the above results suggest that mutations in genes associated with magnesium homeostasis are common in tumors.

### Methylation Pattern of Magnesium Homeostasis-Associated Hub Genes and its Correlation with Gene Expression

**Fig. 3:**
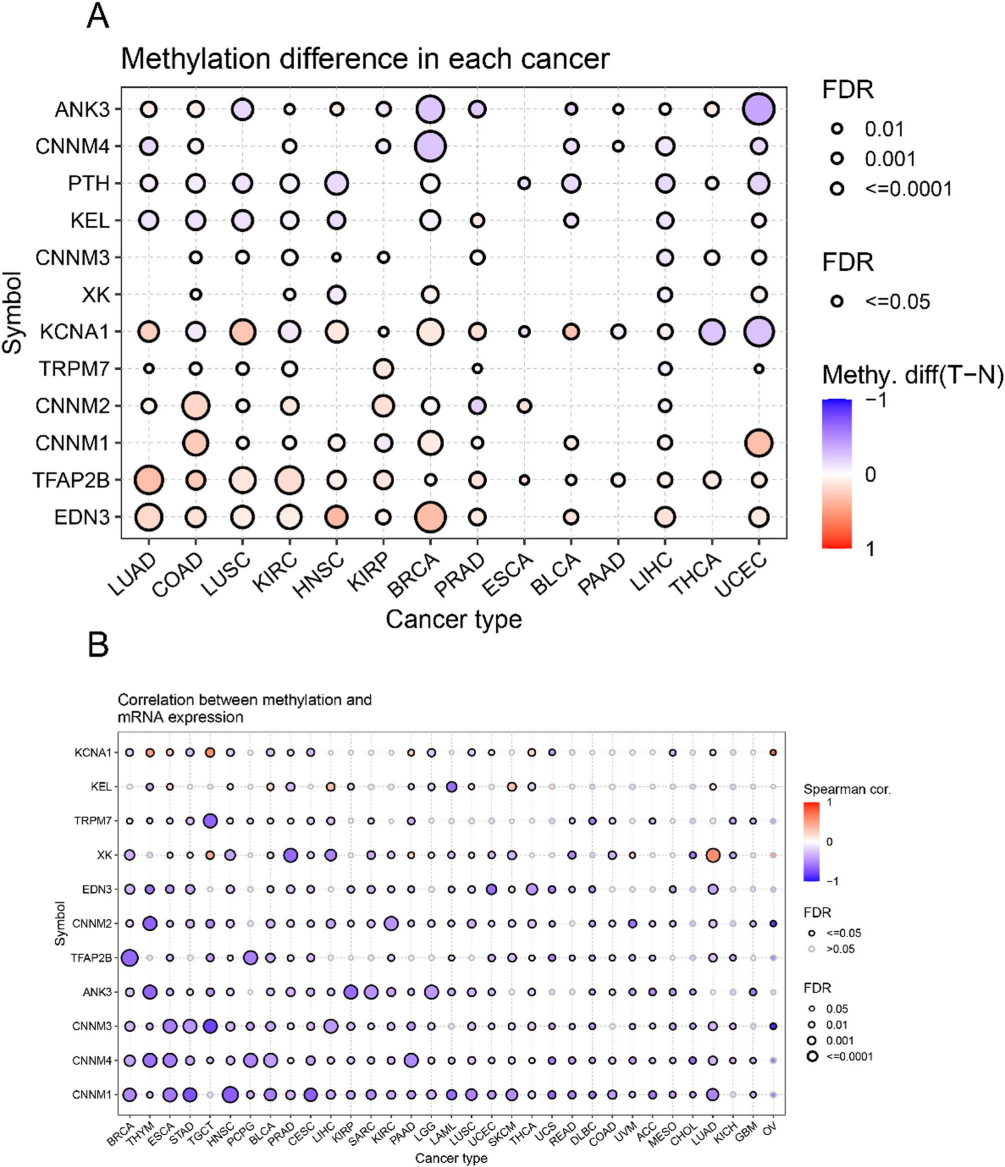
Methylation pattern in magnesium homeostasis-associated hub genes in pan-cancer. **A** Methylation levels of different magnesium homeostasis-associated hub genes in normal and cancer tissues. Red indicates high methylation levels, and purple indicates low methylation levels. **B** Heat map correlating the level of methylation of magnesium homeostasis-associated hub genes with mRNA expression. The larger the dot, the stronger the correlation.dots with black boundaries represent FDR less than 0.05, and larger dots represent smaller FDR

Genomic expression is influenced by DNA methylation. To explore the regulatory mechanisms of MHGs, we evaluated the differences among 14 cancer types in the TCGA database and analyzed the methylation of MHGs in different cancer tissues. The results showed that the methylation levels of MHGs were highly heterogeneous across different cancers, with the majority of MHGs being highly methylated. *EDN3*, *TFAB2B* showed high levels of methylation in almost all cancers, while *CNNM1*, *CNNM2* and *KCNA1* showed high levels of methylation in at least half (7 or more) of the cancers (Fig. 3A). Degree of DNA methylation was correlated with gene expression was negatively correlated, which may account for the differential expression of these MHGs. To confirm the above speculation, we examined the correlation between the methylation pattern and mRNA expression of MHGs. We found that the degree of DNA methylation of MHGs was negatively correlated with gene expression in most cancers (Fig. 3B).

### Drug Sensitivity Analysis

**Fig. 4:**
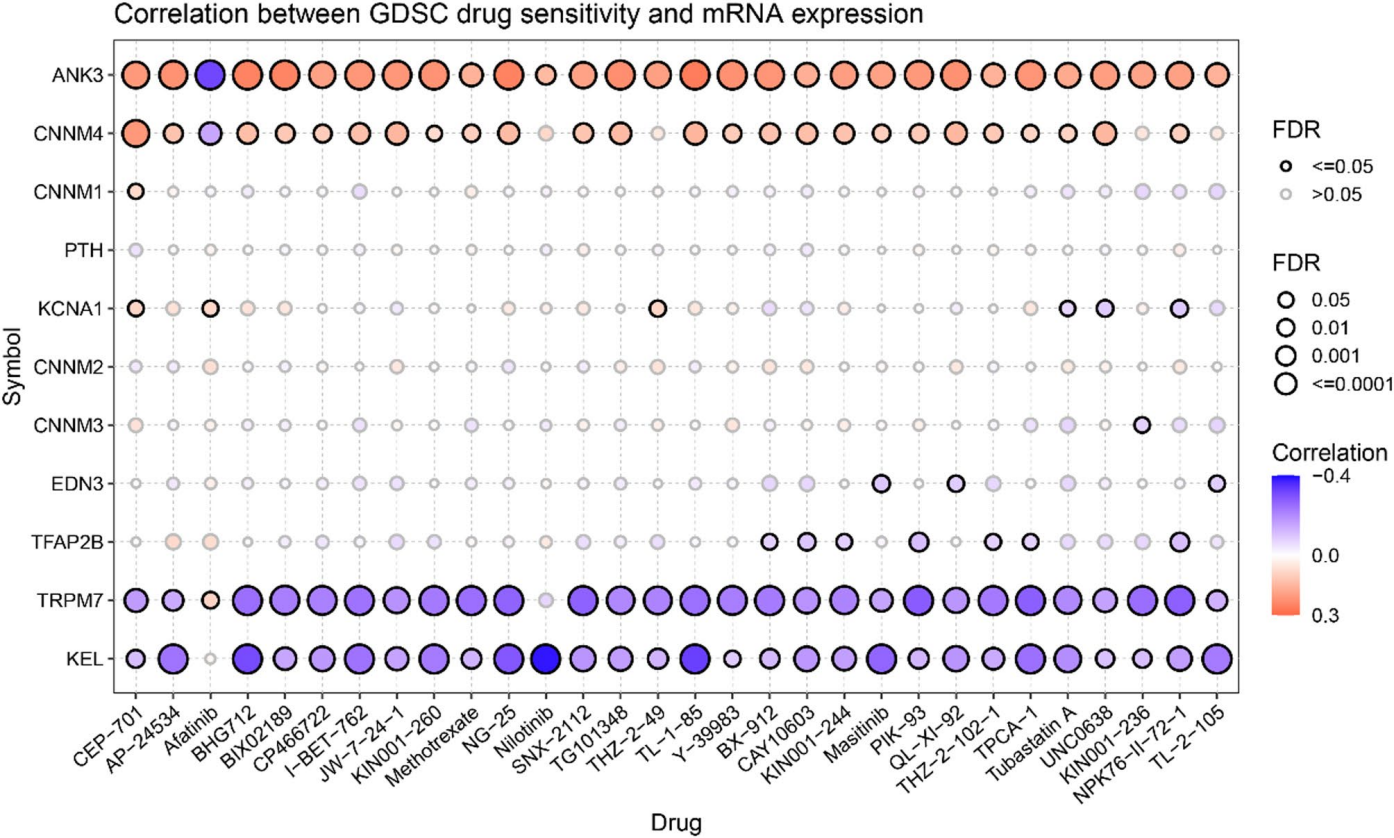
Drug sensitivity analysis of magnesium homeostasis-associated hub genes

Genomic mutations affect the clinical response to chemotherapy and targeted therapies [22]. To understand the effect of MHGs expression on drug response, we analyzed the gene expression profiles of cancer cell lines from the drug sensitivity genomics in the cancer database. We analyzed the correlation between drug sensitivity and gene expression and found that drug sensitivity was strongly correlated with the expression level of MHGs. The results showed that the drug sensitivity of 29 antitumor drugs, except afatinib, was significantly and positively correlated with the expression of ANK3. Similarly, the drug sensitivity of 25 antitumor drugs was positively correlated with the expression of CNNM4, except for afatinib, which was significantly negatively correlated. Conversely, the drug sensitivity of 29 antitumor drugs was significantly negatively correlated with the expression of KEL, except for afatinib, and the drug sensitivity of 28 antitumor drugs was significantly negatively correlated with the expression of TRPM7 (Fig. 4). This information helps to investigate the relationship between anti-cancer drugs and magnesium homeostasis and how this relationship affects the treatment of patients with rare mutations. In conclusion, magnesium homeostasis is critical for cancer development and progression, and maintaining magnesium homeostasis may be a potential tool for cancer therapy.

### Differences in the Magnesium Homeostasis Score and its Correlation with Tumor Stage

**Fig. 5:**
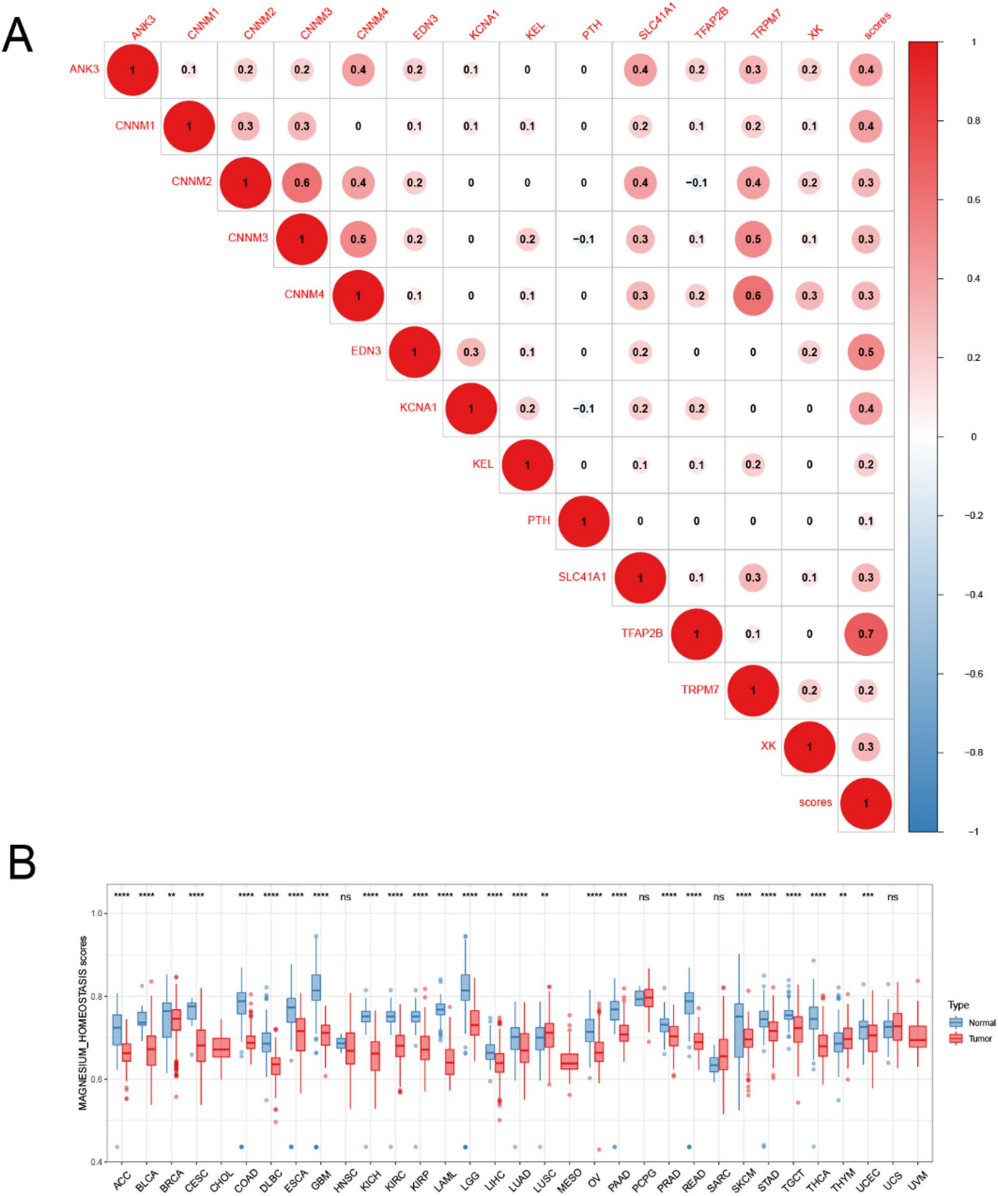
Magnesium homeostasis score in different tissues and cancer stages. **A** Heat map of correlations between the expression of magnesium homeostasis genes and magnesium homeostasis score. The numbers in the circles represent correlation coefficients. **B** Differences in magnesium homeostasis score between tumor and normal tissues. Red represents The Cancer Genome Atlas (TCGA) tumor tissue, and blue represents normal tissue. *P < 0.05, **P < 0.01, ***P < 0.001

To better assess the state of magnesium homeostasis in tumor tissues, we determined a magnesium homeostasis score (MHS). The MHS significantly positively correlated with MHG expression, indicating that the magnesium homeostasis score represents the degree of magnesium homeostasis (*P* < 0.05, Fig. 5A). Simultaneously, we found a positive trend in the expression among most MHGs, for example, TRPM7 showed a significant positive correlation with the gene expression of CNNM3 and CNNM4. Next, we combined the The Cancer Genome Atlas (TCGA) and GETx databases to analyze the difference in MHS between cancer and normal tissues and observed that MHS was almost significantly lower in all 33 cancer types, except for LUSC and UCS. Moreover, the MHS was decreased in stage III/IV compared to stage I/II cancer in BLCA (*P* = 0.007) and THCA (*P* = 0.018) (Supplementary Fig. 2). Therefore, we can infer that MHS plays a role in inhibiting tumor progression.

### Effects of Magnesium Homeostasis on Cancer Prognosis

**Fig. 6:**
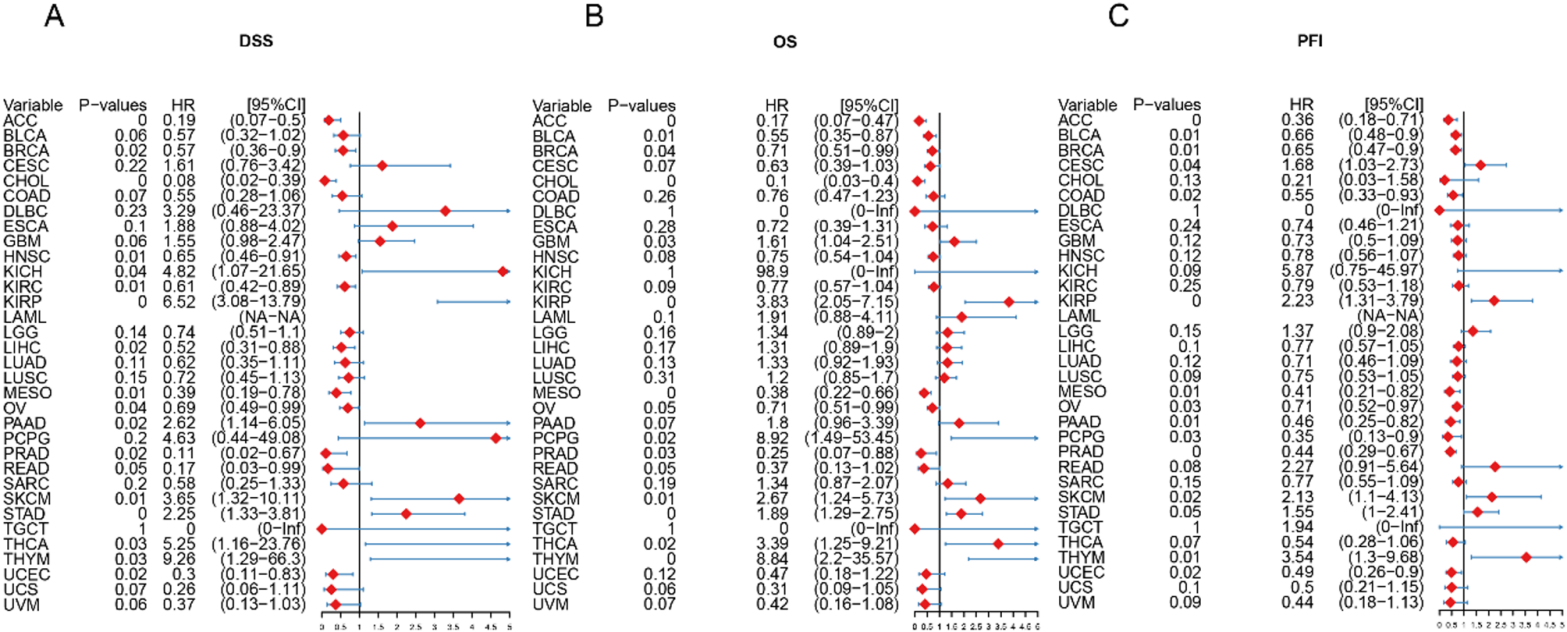
Prognostic value of magnesium homeostasis score. **A** Effect of magnesium homeostasis score on disease-specific survival (DSS). **B** Effect of magnesium homeostasis score on overall survival (OS). **C** Effect of magnesium homeostasis on progression-free interval (PFI). *P < 0.05

For disease-specific survival (DSS), higher magnesium levels had a better prognosis in more tumors (Supplementary Fig. 5), such as ACC (*P* = 0.001, HR = 0.19), LIHC (*P* = 0.016, HR = 0.052), BRCA (*P* = 0.016, HR = 0.57), HNSC (*P* = 0.013, HR = 0.65), READ (*P* = 0.049, HR = 0.17), UCEC (*P* = 0.02, HR = 0.3), CHOL (*P* = 0.001, HR = 0.08), KIRC (*P* = 0.011, HR = 0.61), MESO (*P* = 0.008, HR = 0.39) and OV (*P* = 0.042, HR = 0.69) (Supplementary Fig. 3) Meanwhile, we also found that high magnesium levels in ACC (*P* < 0.001, HR = 0.17), BLCA (*P* = 0.011, HR = 0.55),BRCA(*P* = 0.041, HR = 0.71), MESO(*P* = 0.001, HR = 0.38), and OV(*P* = 0.046, HR = 0.71) were a positive factor for overall survival (OS) (Supplementary Fig. 4). Similar results were found for disease-free progression survival (PFI) (Supplementary Fig. 5). Prognostic analysis of DSS showed that ACC (*P* = 0, HR = 0.19), BRCA (*P* = 0.06, HR = 0.57), CHOL (*P* = 0, HR = 0.08), HNSC (*P* = 0.01, HR = 0.65), KIRC (*P* = 0.01, HR = 0.61), LIHC (*P* = 0.02, HR = 0.52), MESO (*P* = 0.01, HR = 0.39), OV (*P* = 0.04, HR = 0.69), PRAD (*P* = 0.02, HR = 0.11), READ (*P* = 0.05, HR = 0.17), and (*P* = 0.02, HR = 0.3) (Fig. 6A). And Prognostic analysis of OS showed that magnesium homeostasis score was a positive factor for seven cancers, including ACC (*P* = 0, HR = 0.17), BLCA (*P* = 0.01, HR = 0.55), BRCA (*P* = 0.04, HR = 0.71), CHOL (*P* = 0, HR = 0.1), MESO (*P* = 0, HR = 0.38), PRAD (*P* = 0.03, HR = 0.25) and OV (*P* = 0.05, HR = 0.71) (Fig. 6B). The results of PFI were similar to OS, with many tumors showing a significant positive correlation between magnesium homeostasis scores and good prognosis (*P* < 0.05), such as ACC, BLCA, BRCA, COAD, MESO, OV, PAAD, PCPG, PRAD, and UCEC (Fig. 6C). These results suggest that magnesium homeostasis can be used as an independent prognostic marker for a variety of tumors.

### Single-Cell Analysis of the Magnesium Homeostasis Score

**Fig. 7:**
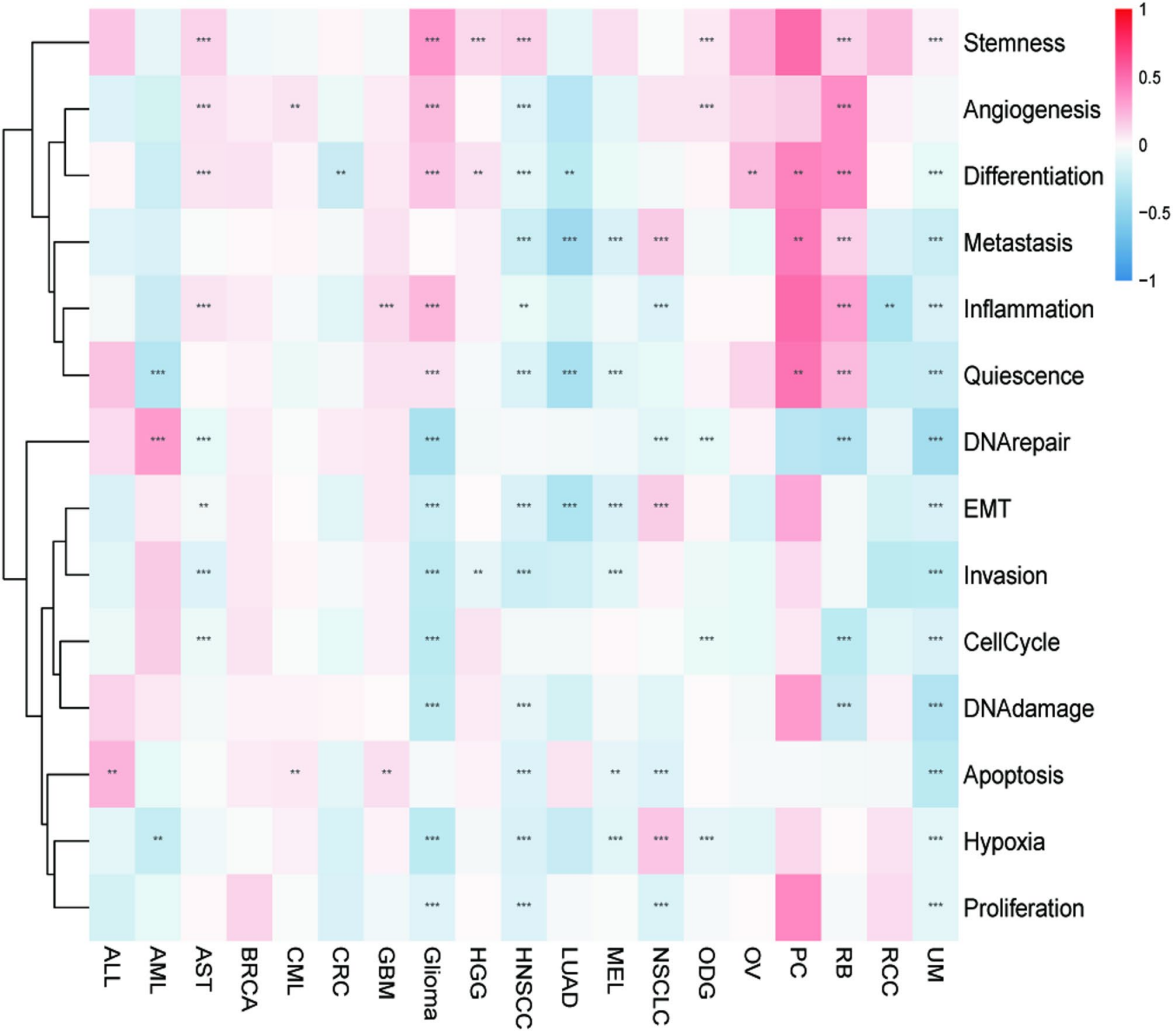
The function of the magnesium homeostasis score at the single-cell level. Correlation of MHS with 14 functional states in different tumors. *P < 0.05, **P < 0.01, ***P < 0.001

The correlation of MHS with 14 functional states of different tumors was analyzed at the single-cell level. Metastasis, quiescence, DNA repair, epithelial-mesenchymal transition (EMT), invasion, cell cycle, DNA damage, apoptosis, hypoxia, and proliferation significantly negatively correlated with the MHGs in most tumor types, indicating that MHGs inhibit the above functions (*P* < 0.05) (Fig. 7). Based on these results, we hypothesized that the death of tumor cells may be related to the inhibition of their cellular functions by magnesium homeostasis.

### Relationship Between the Magnesium Homeostasis Score and TIME

**Fig. 8:**
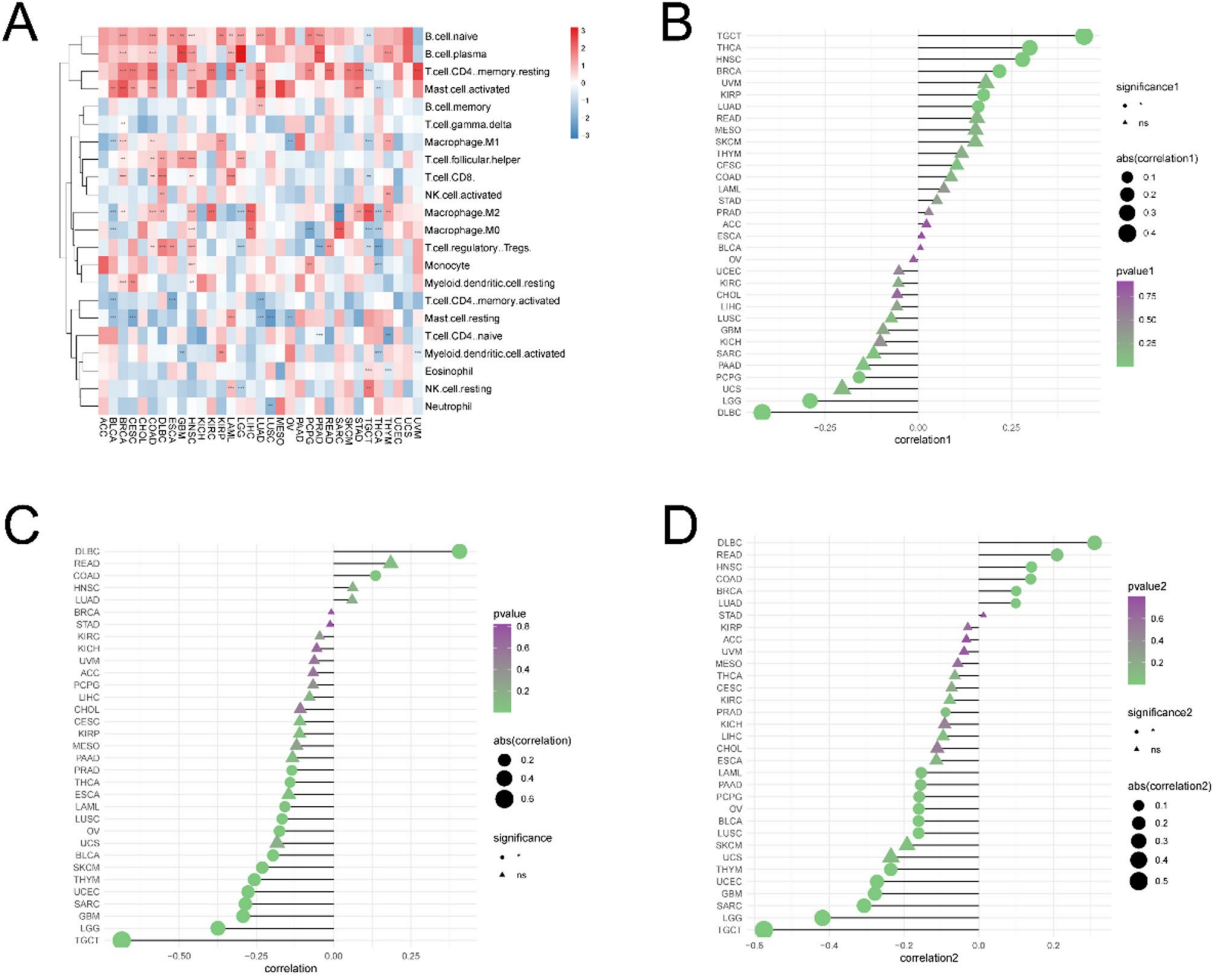
Relationship between the magnesium homeostasis score and the tumor immune microenvironment. **A** Correlation between immune scores of 22 immune cells and MHS. **B** Correlation between MHS and tumor stroma. The larger the point, the larger the absolute value of the correlation coefficient. *P < 0.05. **C** Correlation between MHS and tumor immune score. The larger the point, the larger the absolute value of the correlation coefficient. *P < 0.05. **D** Correlation between MHS and tumor microenvironment. The larger the point, the larger the absolute value of the correlation coefficient. *P < 0.05, **P < 0.01, ***P < 0.001

Tumor purity affects patient prognosis and therapeutic response. Therefore, we analyzed the correlation between the abundance of 22 kinds of immune cells and MHS. MHS significantly positively correlated with the abundance of most immune cells, such as naïve and plasma B cells, resting memory CD4 + T cells, and activated mast cells. MHS also negatively correlated with memory-activated and naïve CD4 + T cells, resting mast cells, activated myeloid dendritic cells, and neutrophils (*P* < 0.05) (Fig. 8A).

MHS also correlated with the tumor microenvironment. There is a complex and significant correlation between magnesium homeostasis score (MHS) and tumor immune microenvironment (TIME). MHS is significantly correlated with the abundance of immune cells, matrix score, immune score and the expression of immune checkpoint molecules, immune activation genes, immunosuppressive genes, chemokines and their receptors. These correlations are different in different tumor types, both positive and negative. MHS significantly positively correlated with the stromal scores in most tumor types, such as TGCT, THCA, HNSC, BRCA, KIRP, and LUAD (Fig. 8B). However, MHS significantly negatively correlated with the immune scores in most cancer types, except for DLBC and COAD (*P* < 0.05) (Fig. 8C), whose correlations were tested individually (Supplementary Fig. 6). Furthermore, MHS significantly positively correlated with tumor immune microenvironment (TIME) for DBLC and negatively correlated with TIME for LGG and TGCT (*P* < 0.05) (Fig. 8D). Thus, the above results indicated that MHS and TIME are closely related.

### Correlation Between the Magnesium Homeostasis Score And immune-related genes

**Fig. 9:**
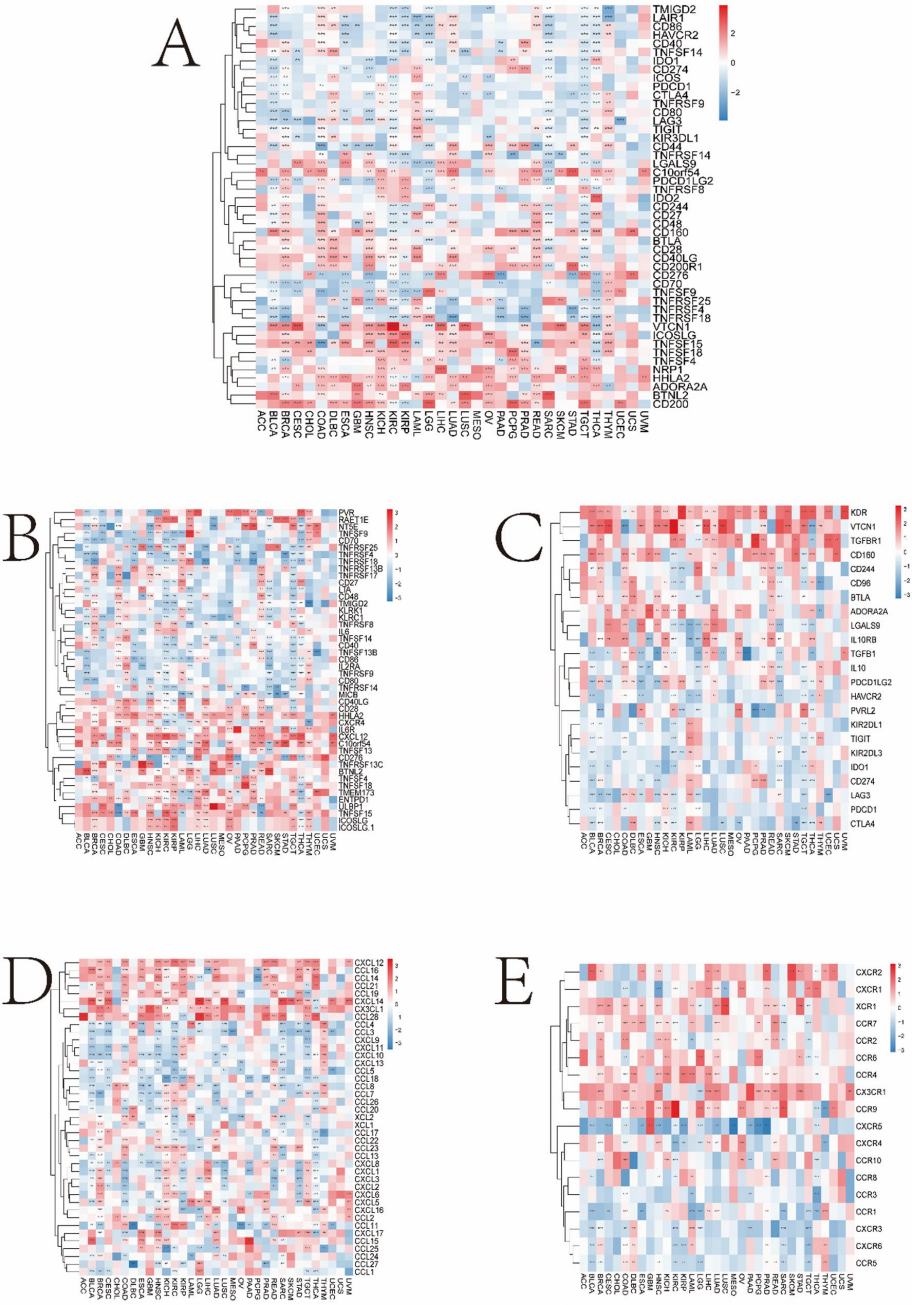
Correlation analyses between the expression of MHGs and immune-related genes. **A** Correlation between MHGs and immune checkpoints in pan-cancer. **B** Correlation between MHGs and immune activation genes in pan-cancer. **C** Correlation between MHGs and different immunosuppressive genes in pan-cancer. **D** Correlation between MHGs and pan-cancer chemokines. **E** Correlation between MHGs and pan-cancer chemokine receptors. *P < 0.05, **P < 0.01, ***P < 0.001

We demonstrated that the MHS was significantly positively correlated with most immune checkpoint molecules such as VTCN1, ICOSLG, TNFSF15, TNFSF18, TNFSF4, NRP1, HHLA2, ADORA2A, BTNL2, and CD200 (*P* < 0.05) (Fig. 9A). In addition, MHS was significantly and positively correlated with the expression of immune activation genes such as TNFRSF13C, BTNL2, TNFSF4, TNFSF18, TMEM173, ENTPD1, ULBP1, TNFSF15, ICOSLG, ICOSLG1, IL6R, and CXCL12, and with TNFRSF4 and CD70 were significantly negatively correlated (*P* < 0.05) (Fig. 9B). MHS were significantly positively correlated with the expression of immunosuppressive genes such as KDR, VTCN1, TGFBR1, and CD160, and significantly negatively correlated with the expression of HAVCR4 (*P* < 0.05) (Fig. 9C).

Furthermore, in most cancers, MHS were significantly positively correlated with the expression of chemokine genes such as CXCL12, CCL16, CCL14, CCL21, CCL19, CXCL14, CX3CL1, and CCL28, and negatively correlated with CCL3, CXCL9, CXCL11, and CXCL10 (*P* < 0.05) (Fig. 9D). Furthermore, in most cancers, MHS were positively correlated with the expression of chemokine receptor genes such as CX3CR1 and CCR9 and negatively correlated with CXCR5 (*P* < 0.05) (Fig. 9E).

### Correlation Between the Magnesium Homeostasis Score and Markers of Immunotherapy Response

**Fig. 10:**
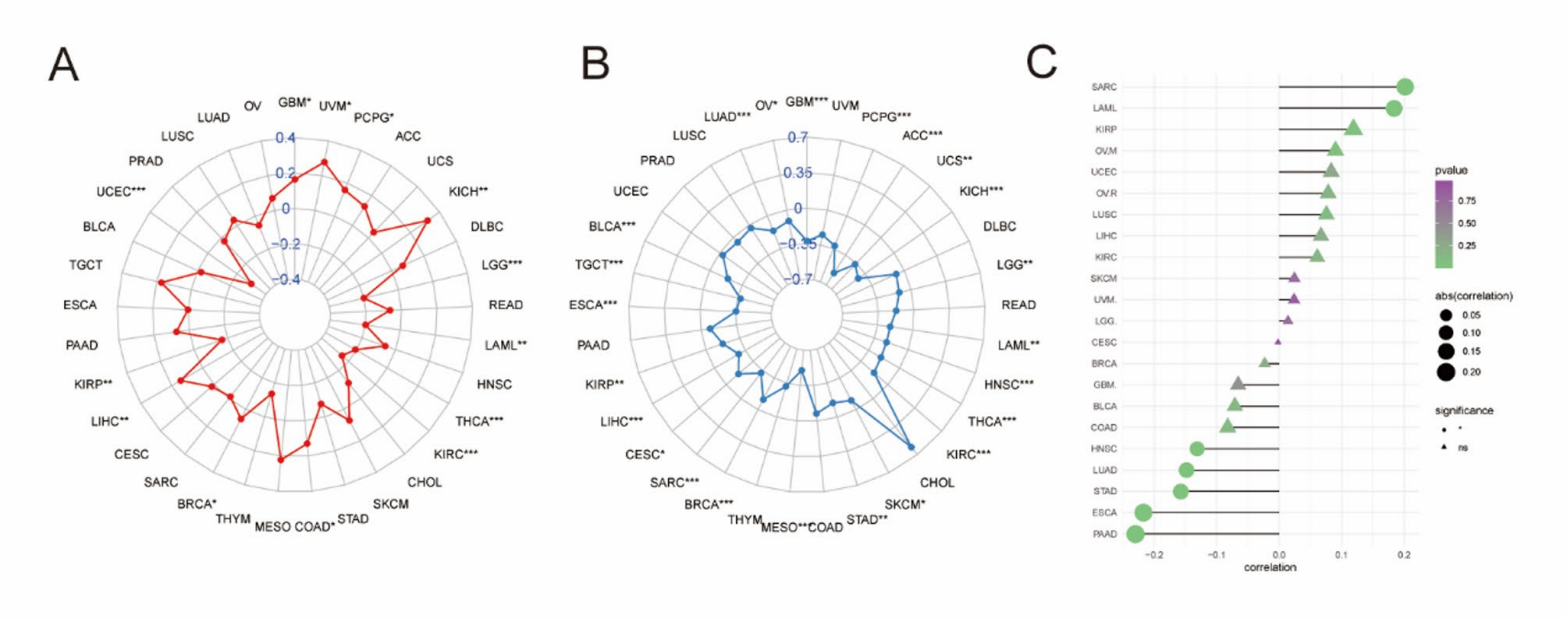
Correlation between the magnesium homeostasis score and the expression of markers related to immunotherapy response. **A** Correlation of MHS scores with microsatellite instability (MSI), **B** tumor mutational burden (TMB), **C** and tumor immune dysfunction and exclusion (TIDE) scores

Magnesium is an important immunomodulator. Therefore, we investigated the role of magnesium homeostasis in immunotherapy. It was found that MHS was closely associated with microsatellite instability (MSI) and tumor mutational load (TMB). In this study, MHS of UVM, KICH and LIHC were significantly positively correlated with MSI, and MHS of UCEC, LGG, LAML, and KIRC were significantly negatively correlated with MSI (Fig. 10A). In addition, MHS scores were negatively correlated with TMB in ACC, KICH, and MESO (Fig. 10B). To test the scientific validity of MHS, we further determined the relationship between MHS and TIDE scores and found that they were significantly positively correlated in SARC and LAML and negatively correlated in PAAD and ESCA (Fig. 10C). Therefore, MHS plays an important role in immunotherapy.

### Analysis of Single-Cell Transcriptional Profile and the Magnesium Homeostasis Score in the KIRC Microenvironment

**Fig. 11:**
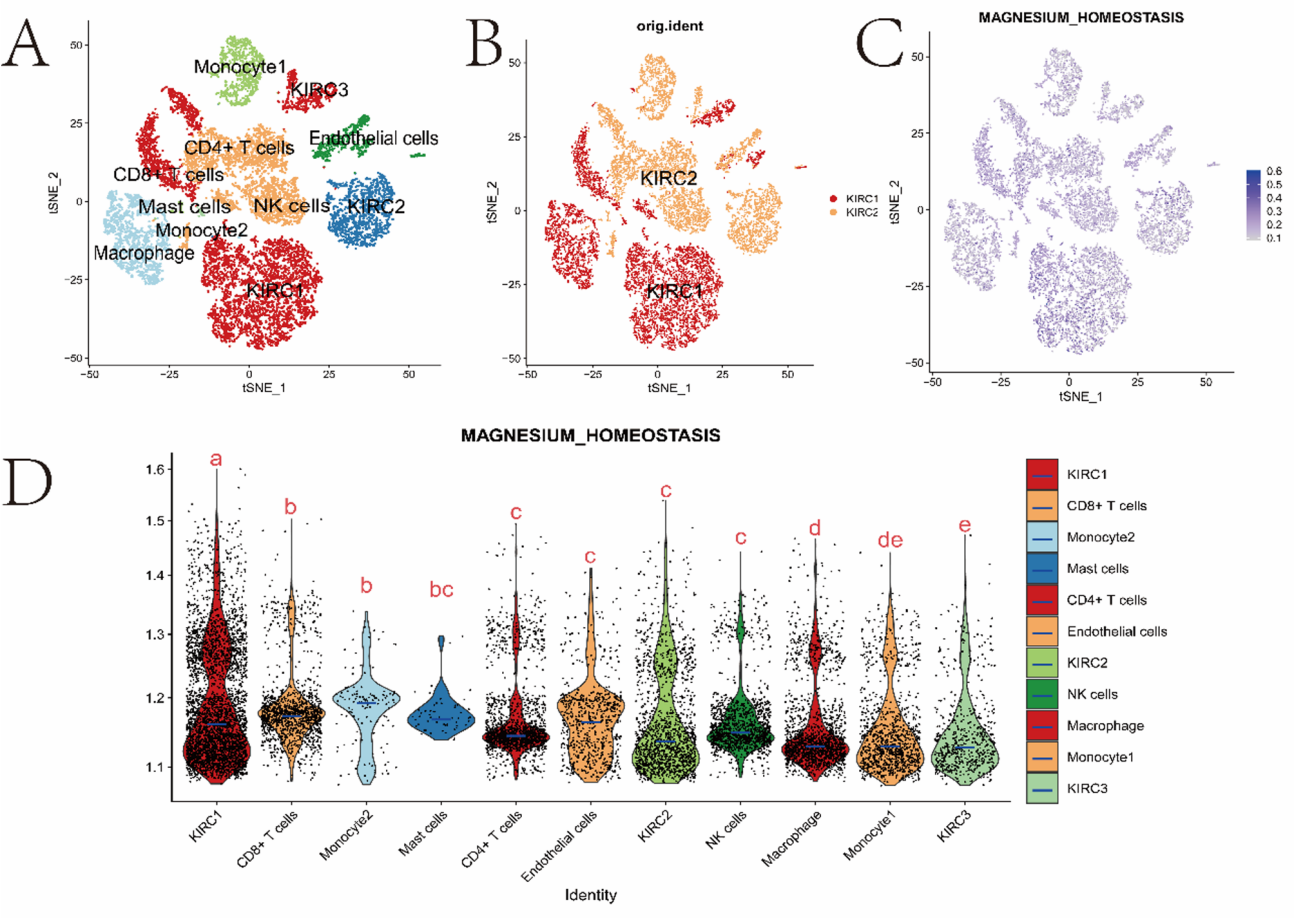
Magnesium homeostasis score analysis based on kidney renal clear cell carcinoma (KIRC) single-cell transcrip-tome atlas. **A** tSEN plot representation of kidney renal clear cell carcinoma (KIRC) samples with 11 distinct cell types. **B** tSEN plot representation of KIRC from two different samples. **C** tSEN plot representation of magnesium homeostasis scores in different cell types. **D** Comparison of MHS in different kidney renal clear cell carcinoma (KIRC) cells

Finally, we performed single-cell sequencing (scRNA-seq) data analysis on two kidney renal clear cell carcinoma (KIRC) samples. We used 13,124 high-quality single-cell transcriptome information for subsequent analysis after quality control using Seurat. Cell clustering analysis based on the tSNE algorithm revealed that the above cells could be classified into 11 clusters, namely KIRC1, KIRC2, KIRC3, Monocyte1, Monocyte2, Macrophage, CD4 + T cells, CD8 + T cells, Mast cells, NK cells, and endothelial cells (Fig. 11A). The marker genes were significantly differentially expressed in different cell populations (Supplementary Fig. 10). We also found that tumor cells from two different sources of KIRC samples contained the same cluster (KIRC3) and unique clusters (KIRC1 and KIRC2) (Fig. 11B). This result indicated the heterogeneity of KIRC cell types. Next, we used ssGSEA to estimate the MHS for KIRC tumor microenvironment cells and compared the differences in magnesium homeostasis score across cell types (Fig. 11C). Interestingly, we found significant differences in the MHS of different cells, and KIRC cells had the highest MHS (Fig. 11D). These results suggest that KIRC cells are significantly active in magnesium metabolism and that magnesium metabolism reflects the cellular heterogeneity of the tumor microenvironment. Therefore, magnesium homeostasis might be critical for regulating the tumor microenvironment.

### Protein Expression of Hub-Genes

**Fig. 12:**
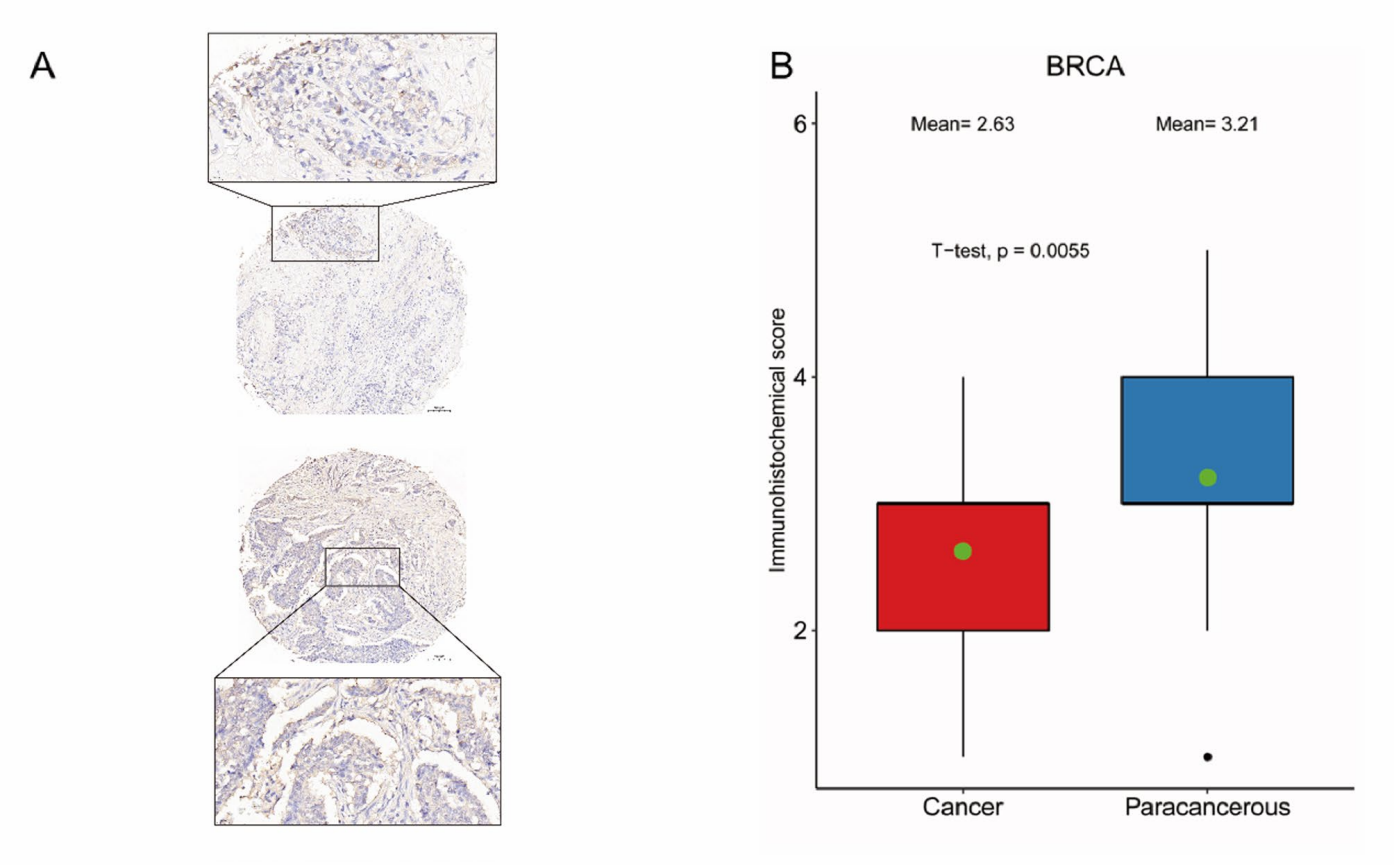
The immunohistochemical staining results and statistical analysis of TRPM7 in breast cancer tissues. **A** Representative immunohistochemical images of breast cancers. **B** Statistical results of TRPM7 expression among 35 pairs of breast cancer and normal samples

The TRPM7, which has the highest 5 degree in the PPI network, was defined as the hub-gene. Moreover, we examined the expression of TRPM7 in 35 pairs of BRCA tissues compared with normal tissues using immunohistochemistry, and the results showed that the expression of TRPM7 in BRCA tissues was significantly lower than that in normal tissues, which was consistent with the results of our differential expression analysis. (Fig. 12, *P* = 0.0005).

## Discussion

Magnesium is a macronutrient and the second most abundant intracellular cation in the body, and magnesium homeostasis is necessary to maintain homeostasis in the body. Studies have shown that tumor cells tend to disrupt magnesium homeostasis [23]. Previously, the magnesium channel protein *TRPM7* has been widely demonstrated to be involved in the development and progression of many cancers, including digestive system cancers, breast cancer and colorectal cancer [24–26]. This suggests that disruption of magnesium homeostasis is a tumor-promoting mechanism, while magnesium homeostasis a tumor-suppressing mechanism that facilitates the initiation of base excision repair in DNA [27]. A higher frequency of hypomagnesemia was associated with shorter survival in patients with advanced ovarian cancer receiving carboplatin-based chemotherapy, and this was confirmed by similar findings in head and neck cancer [28, 29]. There is also evidence that magnesium is a key regulator of cell proliferation [30]. Tumorigenesis occurs when the immune system fails to clear proliferating masses of tumor cells in a timely manner. The relationship between magnesium homeostasis and tumor immunity is not well understood; therefore, in this study, we analyzed and investigated the relationship between tumor progression, prognostic values and immune infiltration at the pan-cancer level, as well as their correlation with the expression of immunotherapeutic markers of magnesium homeostasis. We found a strong correlation between patient responsiveness to immunotherapy and the expression of genes related to magnesium homeostasis. We have observed a robust correlation between patient responsiveness to immunotherapy and the expression of genes associated with magnesium homeostasis. This suggests that magnesium homeostasis may impact the effectiveness of immunotherapy by influencing immune cell function and the tumor microenvironment. Consequently, taking into account a patient’s magnesium homeostasis during the development of an immunotherapy strategy could potentially enhance treatment efficacy and safety.

In this study, we analyzed the correlation of magnesium homeostasis-associated genes with copy number variation, methylation, drug sensitivity, prognostic value, tumor microenvironment, immune-related genes, checkpoints and chemokines and performed single-cell transcriptional profiling and magnesium homeostasis score analysis in the KIRC microenvironment using a comprehensive bioinformatics approach. Single cell analysis of magnesium homeostasis score (MHS) is suitable for a variety of tumors, including but not limited to renal clear cell carcinoma (KIRC), breast cancer, hepatocellular carcinoma (LIHC), colorectal adenocarcinoma (READ), endometrial carcinoma (UCEC), cholangiocarcinoma (CHOL), renal papillary cell carcinoma (KICH), ovarian cancer (OV) and mesothelioma (MESO). In these tumors, MHS is significantly correlated with the expression of immune activation genes, immunosuppressive genes, chemokines and their receptors, indicating that magnesium homeostasis plays an important role in regulating tumor microenvironment and immune response. For example, high MHS in hepatocellular carcinoma (LIHC) is associated with better prognosis, and MHS is positively correlated with MSI, suggesting that it may improve the survival of patients by affecting immune function. Magnesium homeostasis-related genes are significantly differentially expressed in various tumors, and some of them are considered as risk factors for tumor prognosis. In general, the lower the expression of magnesium homeostasis-related genes, the better the prognosis. Subsequently, we measured magnesium homeostasis scores and found that magnesium homeostasis was associated with a better prognosis in cancer patients. The expression of magnesium homeostasis-related genes is closely related to the tumor microenvironment and immune-related genes. Therefore, magnesium homeostasis-related genes could be used as potential prognostic and predictive markers for immunotherapy.

Based on the TCGA database, we found that most magnesium homeostasis-related genes are lowly expressed in most tumors. Examples include ANK3 and CNNM2. Previous studies have shown that ANK3 can promote cell survival by reducing the inhibition of detachment-induced apoptosis, and that ANK3 is a potential therapeutic target for colorectal and lung cancers [31, 32]. Our study found that ANK3 low expression was highly correlated with better cancer disease-specific survival (DSS), overall patient survival (OS) and progression-free survival (PFS) in adrenocortical carcinoma (ACC), colon cancer (COAD), kidney clear cell carcinoma (KIRC) and head and neck squamous cell carcinoma (HNSC). CNNM proteins (including CNNM1 to CNNM4) are widely expressed throughout the human body, and CNNM2 and CNNM4 mediate the efflux of Mg2 + and are responsible for intestinal and renal magnesium reabsorption [33]. CNNM2 is thought to promote cell replication and enhance migration and invasion of cancer cells through the formation of PRL-CNNM complexes [34]. It has also been shown that imbalance in magnesium homeostasis is a biomarker for the diagnosis of colorectal cancer [35]. In our study CNNM2 was also significantly low expressed in colorectal cancer, which is consistent with our findings. Magnesium homeostasis score (MHS) has significant prognostic value in the following cancers: adrenocortical carcinoma (ACC), bladder cancer (BLCA), breast cancer (BRCA), cholangiocarcinoma (CHOL), mesothelioma (MESO) and ovarian cancer (OV). Among these cancers, higher magnesium steady-state scores are associated with better disease-specific survival (DSS), overall survival (OS) and progression-free survival (PFI). Magnesium homeostasis affects tumor development and progress by regulating intracellular energy metabolism, DNA transcription, protein synthesis and immune function. In addition, magnesium homeostasis is also closely related to the expression of immune activation genes in tumor microenvironment, such as TNFRSF13C, BTNL2, TNFSF4 and so on, thus promoting anti-tumor immune response. Therefore, magnesium homeostasis related genes can be used as potential prognostic and predictive markers. The findings that ANK3 and CNNM2 are low expressed in various cancers and associated with better survival outcomes suggest that these genes may serve as useful prognostic biomarkers. By monitoring the expression levels of these genes in cancer patients, clinicians may be able to identify those with a better prognosis. In addition, targeting genes associated with magnesium homeostasis may be a promising cancer treatment strategy. By modulating the expression or activity of these genes, it is possible to alter the tumor microenvironment, disrupt cancer cell proliferation and survival, and ultimately improve patient outcomes.

Subsequently, we unveiled the correlation between magnesium homeostasis scores and the composition of the tumor microenvironment, which encompasses immune cells, cancer-associated fibroblasts, blood vessels, lymphatic endothelial cells, and extracellular matrix components. The tumor microenvironment is widely recognized as a pivotal factor in tumorigenesis and progression. Alterations in magnesium homeostasis closely associated with changes in the tumor microenvironment are intricately linked to tumorigenesis and progression; thus, magnesium homeostasis is believed to exert its influence on tumorigenesis and progression by modulating the tumor microenvironment [10]. Through single-cell sequencing analysis, we observed significant variations in magnesium homeostasis scores among different cell types within the tumor microenvironment; for instance, CD8 + T cells and mast cells exhibited higher scores while monocytes and macrophages displayed lower scores. This observation suggests a potential role of magnesium metabolism in governing the regulation of the tumor microenvironment. In magnesium-deficient mice models, elevated levels of TNF-α and IL-6 total mRNA were detected in proximal colon tissues indicating an impact of low magnesium on cellular inflammatory stress [33]. By advocating for altered magnesium homeostasis status, an increase in CD8 + T cell infiltration along with a decrease in immunosuppressive myeloid-derived suppressor cells (MDSC) can be observed—signifying a modulation towards immune-supporting cells within the immunosuppressive tumor microenvironment [34]. Consequently, targeting genes related to magnesium homeostasis may offer promising therapeutic effects by reshaping the characteristics of the tumor microenvironment—thus laying a solid foundation for personalized treatment strategies for patients with tumors.

Finally, we also revealed the correlation between magnesium homeostasis scores and markers of immunotherapy response. Our results show that magnesium homeostasis score has a significant positive correlation with MSI score and a significant negative correlation with TMB score and TIDE score in a variety of tumors.TMB score, MSI score and TIDE score are currently recognized markers of ICB therapy. TMB is for the total number of non-anonymous mutations per megabase in somatic cells, including shift mutations, insertions, point mutations and deletions [36, 37]. These mutations lead to the production of abnormal proteins, which can function as neoantigens and activate anti-tumor responses [38]. Microsatellite instability (MSI) is caused by a functional defect in DNA mismatch repair in tumor tissue and is a clinically important tumor marker. The TIDE score is a novel and most promising marker of ICB response, and there is growing evidence that the Tide score is more accurate than the MSI and TMB scores in predicting survival outcomes in cancer patients treated with ICB drugs [39]. In summary, the results of this study suggest that genes related to magnesium homeostasis can be an independent prognostic factor for ICB treatment. The present study used an integrated bioinformatics approach to reveal the role of magnesium homeostasis related genes in influencing the prognosis of patients with multiple cancer types. Our findings suggest that magnesium homeostasis plays a key role in regulating tumor microenvironment and immunity and may serve as a prognostic biomarker. However, some limitations remain and further research is needed to consolidate our understanding and translate these findings into clinical practice. The future research direction is to explore the mechanism relationship between magnesium homeostasis and tumor immunity. This may involve studying specific signaling pathways and gene expression changes that are in response to changes in magnesium levels. In addition, rigorous in vitro and in vivo experiments must be conducted to validate our bioinformatics findings. By controlling magnesium levels in cancer cell lines and animal models, we can observe changes in tumor behavior and immune response, further confirming the role of magnesium homeostasis in cancer progression. In conclusion, the current study lays the foundation for further research into the role of magnesium in cancer. By exploring the mechanologic link between magnesium homeostasis and tumor immunity, conducting rigorous in vitro and in vivo experiments, and initiating large-scale clinical trials, we can further consolidate our understanding of this important biological process and translate our findings into improved patient outcomes.

Emerging evidence highlights the critical role of magnesium ion (Mg²⁺) homeostasis in tumor immunity and metastasis, with clinical implications centered on biomarkers and therapeutic strategies: Magnesium Homeostasis Scores (MHS), derived from transporter genes (e.g.: CNNM2, ANK3), may stratify immunotherapy responders and predict metastasis risk in breast cancer and melanoma [40], while Phase I/II trials in immunotherapy-resistant tumors suggest Mg²⁺ supplementation could enhance CD8 + T cell infiltration. Targeting Mg²⁺ transporters—such as CNNM2 inhibitors or TRPM7 activators—may simultaneously block tumor Mg²⁺ uptake and amplify T cell functionality. Combination therapies involving Mg²⁺ modulators (e.g.: ionophores) with anti-PD-1/CTLA-4 agents could reverse the “cold” tumor phenotype, whereas Mg²⁺ chelators (e.g.: EDTA) might improve chemotherapy sensitivity [41]. Current research priorities include validating MHS clinical applicability through spatial transcriptomics, dissecting transporter roles in Mg²⁺ flux and immune evasion via CRISPR knockout models, and identifying Mg²⁺-dependent metabolic pathways in CD8 + T cells using single-cell multiomics [5]. Challenges such as MHS standardization, ensuring transporter-targeted therapy specificity, and balancing systemic versus tumor microenvironment Mg²⁺ levels remain unresolved. Nonetheless, integrating Mg²⁺-related biomarkers and transporter-targeted strategies holds promise for optimizing metabolic-immune crosstalk networks, potentially reshaping cancer treatment paradigms.

## Conclusion

This study is the first comprehensive pan-cancer-level analysis of the role of magnesium homeostasis. The results of this study suggest that magnesium homeostasis plays an important role in tumor immunotherapy and can act as an immune marker. Therefore, therapeutic approaches targeting magnesium homeostasis may be a novel breakthrough strategy for cancer treatment. However, there are also certain limitations to this study, and clinical validation of the immunotherapy effect of magnesium homeostasis at the pan-cancer level is still required in the future.

## Supplementary Information

Below is the link to the electronic supplementary material.

Supplementary Material 1.

## Data Availability

The data supporting the findings of this study are deposited in TCGA and GEO databases. The single-cell sequencing datasets can be found in online repositories of GEO (GSE152938).

## References

[1] Bray F, Ferlay J, Soerjomataram I, Siegel RL, Torre LA, Jemal A. Global cancer statistics 2018: GLOBOCAN estimates of incidence and mortality worldwide for 36 cancers in 185 countries. CA Cancer J Clin. 2018;68:394–424. 10.3322/caac.21492. 30207593 10.3322/caac.21492

[2] Hassannia B, Vandenabeele P, Vanden Berghe T. Targeting ferroptosis to iron out cancer. Cancer Cell. 2019;35:830–49. 10.1016/j.ccell.2019.04.002. 31105042 10.1016/j.ccell.2019.04.002

[3] Tsvetkov P, Coy S, Petrova B, Dreishpoon M, Verma A, Abdusamad M, Rossen J, Joesch-Cohen L, Humeidi R, Spangler RD, Eaton JK, Frenkel E, Kocak M, Corsello SM, Lutsenko S, Kanarek N, Santagata S, Golub TR. Copper induces cell death by targeting lipoylated TCA cycle proteins. Science. 2022;375:1254–61. 10.1126/science.abf0529. 35298263 10.1126/science.abf0529 PMC9273333

[4] Pickering G, Mazur A, Trousselard M, Bienkowski P, Yaltsewa N, Amessou M, Noah L, Pouteau E. Magnesium status and stress: the vicious circle concept revisited. Nutrients. 2020. 10.3390/nu12123672. 33260549 10.3390/nu12123672 PMC7761127

[5] de Baaij JH, Hoenderop JG, Bindels RJ. Magnesium in man: implications for health and disease. Physiol Rev. 2015;95:1–46. 10.1152/physrev.00012.2014. 25540137 10.1152/physrev.00012.2014

[6] Maier JA, Castiglioni S, Locatelli L, Zocchi M, Mazur A. Magnesium and inflammation: advances and perspectives. Semin Cell Dev Biol. 2021;115:37–44. 10.1016/j.semcdb.2020.11.002. 33221129 10.1016/j.semcdb.2020.11.002

[7] Yokoyama T, Fujii S, Ostermann A, Schrader TE, Nabeshima Y, Mizuguchi M. Neutron crystallographic analysis of the nucleotide-binding domain of Hsp72 in complex with ADP. IUCrJ. 2022;9:562–72. 10.1107/s2052252522006297. 36071806 10.1107/S2052252522006297 PMC9438496

[8] Murphy E. Mysteries of magnesium homeostasis. Circ Res. 2000;86:245–8. 10.1161/01.res.86.3.245. 10679471 10.1161/01.res.86.3.245

[9] Zinov’eva VN, Iezhitsa IN, Spasov AA. Magnesium homeostasis: mechanisms and inherited disorders. Biomed Khim. 2007;53:683–704. 18323153

[10] Kaplinsky C, Alon US. Magnesium homeostasis and hypomagnesemia in children with malignancy. Pediatr Blood Cancer. 2013;60:734–40. 10.1002/pbc.24460. 23303583 10.1002/pbc.24460

[11] Ryazanova LV, Rondon LJ, Zierler S, Hu Z, Galli J, Yamaguchi TP, Mazur A, Fleig A, Ryazanov AG. TRPM7 is essential for Mg(2+) homeostasis in mammals. Nat Commun. 2010;1(1): 109. 10.1038/ncomms1108. 21045827 10.1038/ncomms1108 PMC3060619

[12] Castiglioni S, Maier JA. Magnesium and cancer: a dangerous liason. Magnes Res. 2011;24:S92–100. 10.1684/mrh.2011.0285. 21933757 10.1684/mrh.2011.0285

[13] Su C, Lv Y, Lu W, Yu Z, Ye Y, Guo B, Liu D, Yan H, Li T, Zhang Q, Cheng J, Mo Z. Single-cell RNA sequencing in multiple pathologic types of renal cell carcinoma revealed novel potential tumor-specific markers. Front Oncol. 2021;11: 719564. 10.3389/fonc.2021.719564. 34722263 10.3389/fonc.2021.719564 PMC8551404

[14] Lu Y, Feng Y, McNally A, Zong Z. Occurrence of colistin-resistant hypervirulent *Klebsiella variicola*. J Antimicrob Chemother. 2018;73(11):3001–4. 10.1093/jac/dky301. 30060219 10.1093/jac/dky301

[15] Wenzel TJ, Gates EJ, Ranger AL, Klegeris A. Short-chain fatty acids (SCFAs) alone or in combination regulate select immune functions of microglia-like cells. Mol Cell Neurosci. 2020;105: 103493. 10.1016/j.mcn.2020.103493. 32333962 10.1016/j.mcn.2020.103493

[16] Speir ML, Bhaduri A, Markov NS, Moreno P, Nowakowski TJ, Papatheodorou I, Pollen AA, Raney BJ, Seninge L, Kent WJ, Haeussler M. UCSC cell browser: visualize your single-cell data. Bioinformatics. 2021;37:4578–80. 10.1093/bioinformatics/btab503. 34244710 10.1093/bioinformatics/btab503 PMC8652023

[17] Jäger E, Jäger D, Knuth A. CTL-defined cancer vaccines: perspectives for active immunotherapeutic interventions in minimal residual disease. Cancer Metastasis Rev. 1999;18:143–50. 10.1023/a:1006220707618. 10505552 10.1023/a:1006220707618

[18] Huang R, Mao M, Lu Y, Yu Q, Liao L. A novel immune-related genes prognosis biomarker for melanoma: associated with tumor microenvironment. Aging. 2020;12:6966–80. 10.18632/aging.103054. 32310824 10.18632/aging.103054 PMC7202520

[19] Liu P, Jiang W, Zhao J, Zhang H. Integrated analysis of genome–wide gene expression and DNA methylation microarray of diffuse large B–cell lymphoma with TET mutations. Mol Med Rep. 2017;16:3777–82. 10.3892/mmr.2017.7058. 28731140 10.3892/mmr.2017.7058 PMC5646955

[20] Albaradei S, Thafar M, Alsaedi A, Van Neste C, Gojobori T, Essack M, Gao X. Machine learning and deep learning methods that use omics data for metastasis prediction. Comput Struct Biotechnol J. 2021;19:5008–18. 10.1016/j.csbj.2021.09.001. 34589181 10.1016/j.csbj.2021.09.001 PMC8450182

[21] Lei T, Qian H, Lei P, Hu Y. Ferroptosis-related gene signature associates with immunity and predicts prognosis accurately in patients with osteosarcoma. Cancer Sci. 2021;112:4785–98. 10.1111/cas.15131. 34506683 10.1111/cas.15131 PMC8586685

[22] Miranda Furtado CL, Dos Santos Luciano MC, Silva Santos RD, Furtado GP, Moraes MO, Pessoa C. Epidrugs: targeting epigenetic marks in cancer treatment. Epigenetics. 2019;14:1164–76. 10.1080/15592294.2019.1640546. 31282279 10.1080/15592294.2019.1640546 PMC6791710

[23] Hanahan D, Weinberg RA. Hallmarks of cancer: the next generation. Cell. 2011;144:646–74. 10.1016/j.cell.2011.02.013. 21376230 10.1016/j.cell.2011.02.013

[24] Auwercx J, Rybarczyk P, Kischel P, Dhennin-Duthille I, Chatelain D, Sevestre H, Van Seuningen I, Ouadid-Ahidouch H, Jonckheere N, Gautier M. Mg(2+) transporters in digestive cancers. Nutrients. 2021. 10.3390/nu13010210. 33450887 10.3390/nu13010210 PMC7828344

[25] Bezerra DLC, Mendes PMV, Melo SRS, Dos Santos LR, Santos RO, Vieira SC, Henriques GS, Freitas B, Marreiro DDN. Hypomagnesemia and its relationship with oxidative stress markers in women with breast cancer. Biol Trace Elem Res. 2021;199:4466–74. 10.1007/s12011-021-02579-4. 33443661 10.1007/s12011-021-02579-4

[26] Pugliese D, Armuzzi A, Castri F, Benvenuto R, Mangoni A, Guidi L, Gasbarrini A, Rapaccini GL, Wolf FI, Trapani V. TRPM7 is overexpressed in human IBD-related and sporadic colorectal cancer and correlates with tumor grade. Dig Liver Dis. 2020;52:1188–94. 10.1016/j.dld.2020.05.027. 32505565 10.1016/j.dld.2020.05.027

[27] Adhikari S, Toretsky JA, Yuan L, Roy R. Magnesium, essential for base excision repair enzymes, inhibits substrate binding of N-methylpurine-DNA glycosylase. J Biol Chem. 2006;281:29525–32. 10.1074/jbc.M602673200. 16901897 10.1074/jbc.M602673200

[28] Liu W, Qdaisat A, Soliman PT, Ramondetta L, Lopez G, Narayanan S, Zhou S, Cohen L, Bruera E, Yeung SJ. Hypomagnesemia and survival in patients with ovarian cancer who received chemotherapy with carboplatin. Oncologist. 2019;24:e312–7. 10.1634/theoncologist.2018-0465. 30940743 10.1634/theoncologist.2018-0465 PMC6656478

[29] Liu W, Qdaisat A, Ferrarotto R, Fuller CD, Guo M, Meyer LA, Narayanan S, Lopez G, Cohen L, Bruera E, Hanna EY, Yeung SJ. Hypomagnesemia and survival in patients with head and neck cancers who received primary concurrent chemoradiation. Cancer. 2021;127:528–34. 10.1002/cncr.33283. 33085092 10.1002/cncr.33283 PMC8884478

[30] Trapani V, Wolf FI. Dysregulation of Mg(2+) homeostasis contributes to acquisition of cancer hallmarks. Cell Calcium. 2019;83: 102078. 10.1016/j.ceca.2019.102078. 31493712 10.1016/j.ceca.2019.102078

[31] Menyhart O, Kakisaka T, Pongor LS, Uetake H, Goel A, Győrffy B. Uncovering potential therapeutic targets in colorectal cancer by deciphering mutational status and expression of druggable oncogenes. Cancers. 2019. 10.3390/cancers11070983. 31337155 10.3390/cancers11070983 PMC6679198

[32] Shriwash N, Singh P, Arora S, Ali SM, Ali S, Dohare R. Identification of differentially expressed genes in small and non-small cell lung cancer based on meta-analysis of mRNA. Heliyon. 2019;5:e01707. 10.1016/j.heliyon.2019.e01707. 31338439 10.1016/j.heliyon.2019.e01707 PMC6580189

[33] Huang Y, Jin F, Funato Y, Xu Z, Zhu W, Wang J, Sun M, Zhao Y, Yu Y, Miki H, Hattori M. Structural basis for the Mg(2+) recognition and regulation of the CorC Mg(2+) transporter. Sci Adv. 2021. 10.1126/sciadv.abe6140. 33568487 10.1126/sciadv.abe6140 PMC7875539

[34] Gimenez-Mascarell P, Oyenarte I, Hardy S, Breiderhoff T, Stuiver M, Kostantin E, Diercks T, Pey AL, Ereno-Orbea J, Martinez-Chantar ML, Khalaf-Nazzal R, Claverie-Martin F, Muller D, Tremblay ML, Martinez-Cruz LA. Structural basis of the oncogenic interaction of phosphatase PRL-1 with the magnesium transporter CNNM2. J Biol Chem. 2017;292:786–801. 10.1074/jbc.M116.759944. 27899452 10.1074/jbc.M116.759944 PMC5247653

[35] Schiroli D, Marraccini C, Zanetti E, Ragazzi M, Gianoncelli A, Quartieri E, Gasparini E, Iotti S, Baricchi R, Merolle L. Imbalance of Mg homeostasis as a potential biomarker in colon cancer. Diagnostics. 2021. 10.3390/diagnostics11040727. 33923883 10.3390/diagnostics11040727 PMC8073761

[36] McNamara MG, Jacobs T, Lamarca A, Hubner RA, Valle JW, Amir E. Impact of high tumor mutational burden in solid tumors and challenges for biomarker application. Cancer Treat Rev. 2020;89: 102084. 10.1016/j.ctrv.2020.102084. 32738738 10.1016/j.ctrv.2020.102084

[37] Yarchoan M, Hopkins A, Jaffee EM. Tumor mutational burden and response rate to PD-1 inhibition. N Engl J Med. 2017;377:2500–1. 10.1056/NEJMc1713444. 29262275 10.1056/NEJMc1713444 PMC6549688

[38] Goodman AM, Kato S, Bazhenova L, Patel SP, Frampton GM, Miller V, Stephens PJ, Daniels GA, Kurzrock R. Tumor mutational burden as an independent predictor of response to immunotherapy in diverse cancers. Mol Cancer Ther. 2017;16:2598–608. 10.1158/1535-7163.MCT-17-0386. 28835386 10.1158/1535-7163.MCT-17-0386 PMC5670009

[39] Wang Y, Tong Z, Zhang W, Zhang W, Buzdin A, Mu X, Yan Q, Zhao X, Chang HH, Duhon M, Zhou X, Zhao G, Chen H, Li X. Fda-approved and emerging next generation predictive biomarkers for immune checkpoint inhibitors in cancer patients. Front Oncol. 2021;11: 683419. 10.3389/fonc.2021.683419. 34164344 10.3389/fonc.2021.683419 PMC8216110

[40] Maeda K, Anand K, Chiapparino A, Kumar A, Poletto M, Kaksonen M, Gavin AC. Interactome map uncovers phosphatidylserine transport by oxysterol-binding proteins. Nature. 2013;501:257–61. 10.1038/nature12430. 23934110 10.1038/nature12430

[41] Kramer CK, Retnakaran R, Zinman B. Insulin and insulin analogs as antidiabetic therapy: a perspective from clinical trials. Cell Metab. 2021;33:740–7. 10.1016/j.cmet.2021.03.014. 33826916 10.1016/j.cmet.2021.03.014

