# Supplementary material for: Pan-Cancer Landscape of Magnesium Homeostasis: Bulk Omics Research and Single-Cell Sequencing Validation
Source: Biol Proced Online. 2025 Oct 31;27:42. doi: 10.1186/s12575-025-00294-1 (PMC12576994; doi:10.1186/s12575-025-00294-1)
Supplement: Supplementary file 1 — Supplementary Material 1. [file 12575_2025_294_MOESM1_ESM.docx]

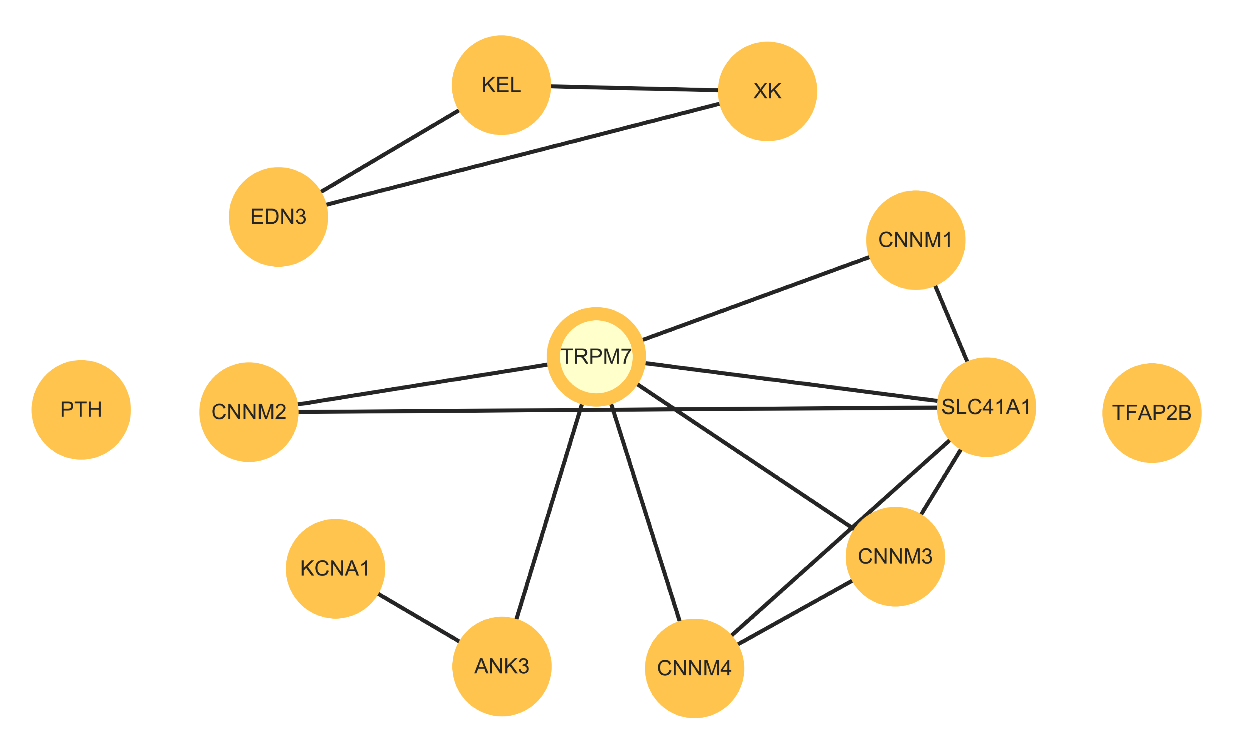


**Supplementary Figure 1** Identification of magnesium homeostasis-associated genes. Network map of magnesium homeostasis-associated genes.

**
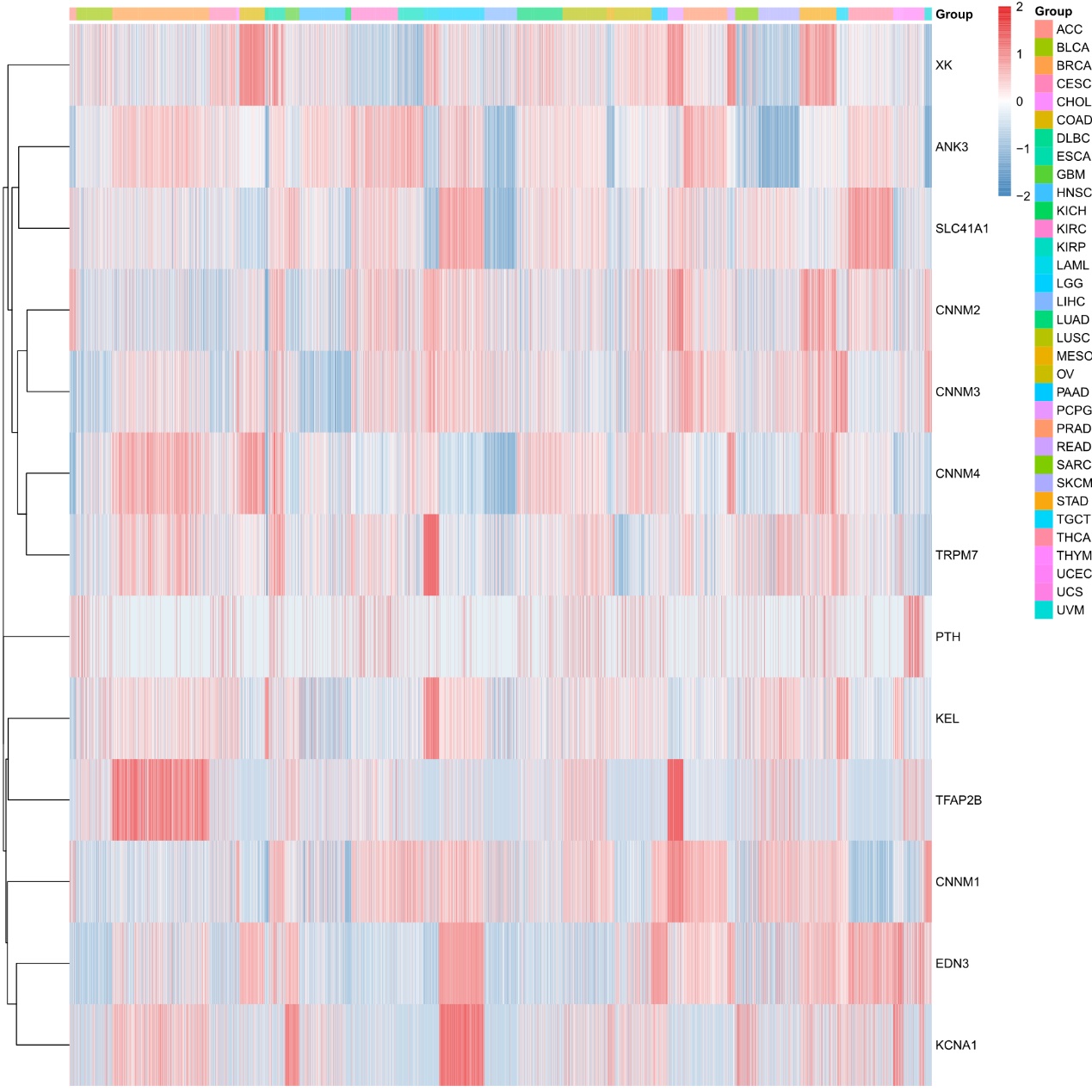
**

**Supplementary Figure 2** Relationship between magnesium homeostasis score and tumor stage based on WHO (World Health Organization).

**
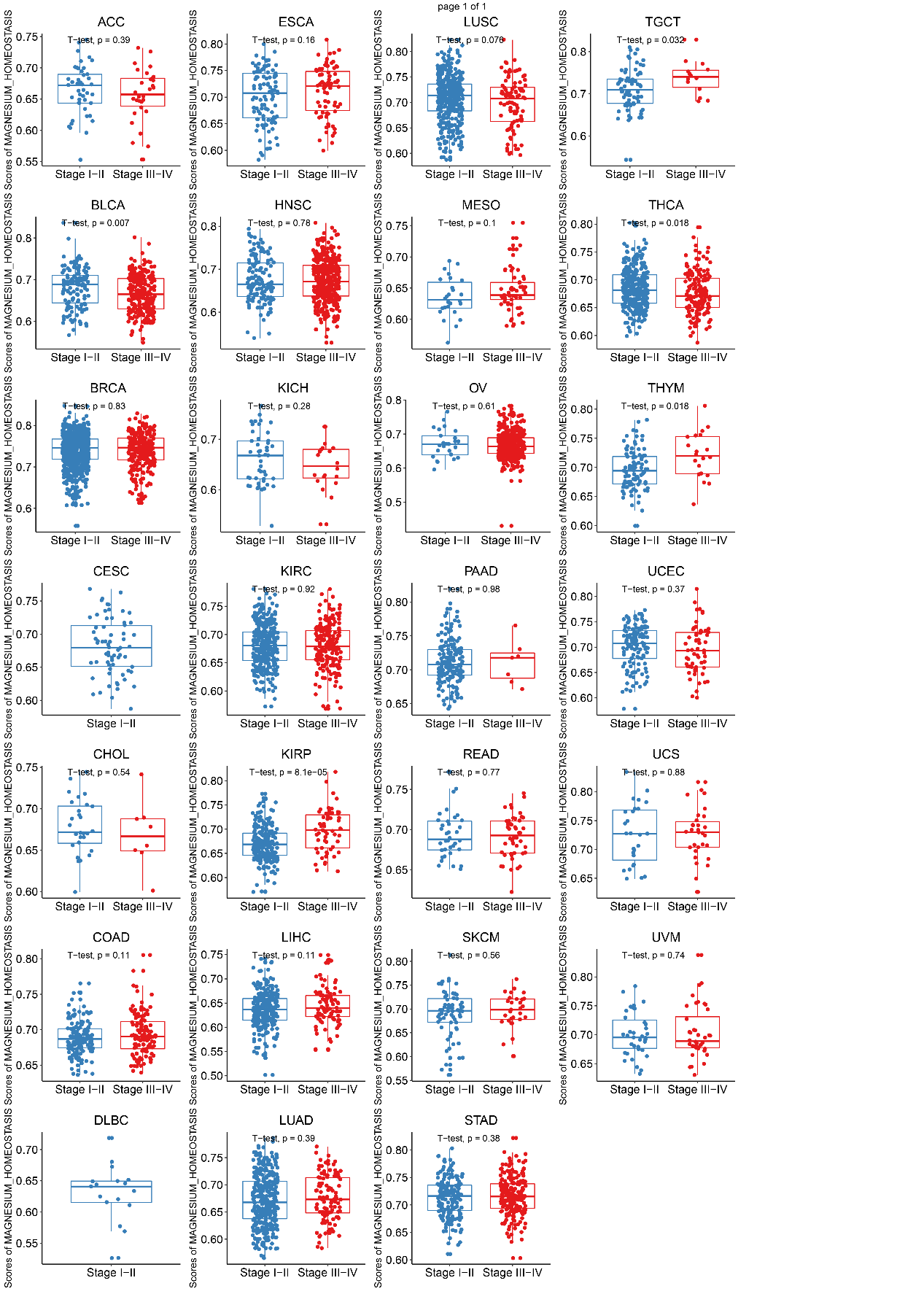
**

**Supplementary Figure 3** Effect of magnesium homeostasis on disease-specificoverall survival (DSSOS).

**
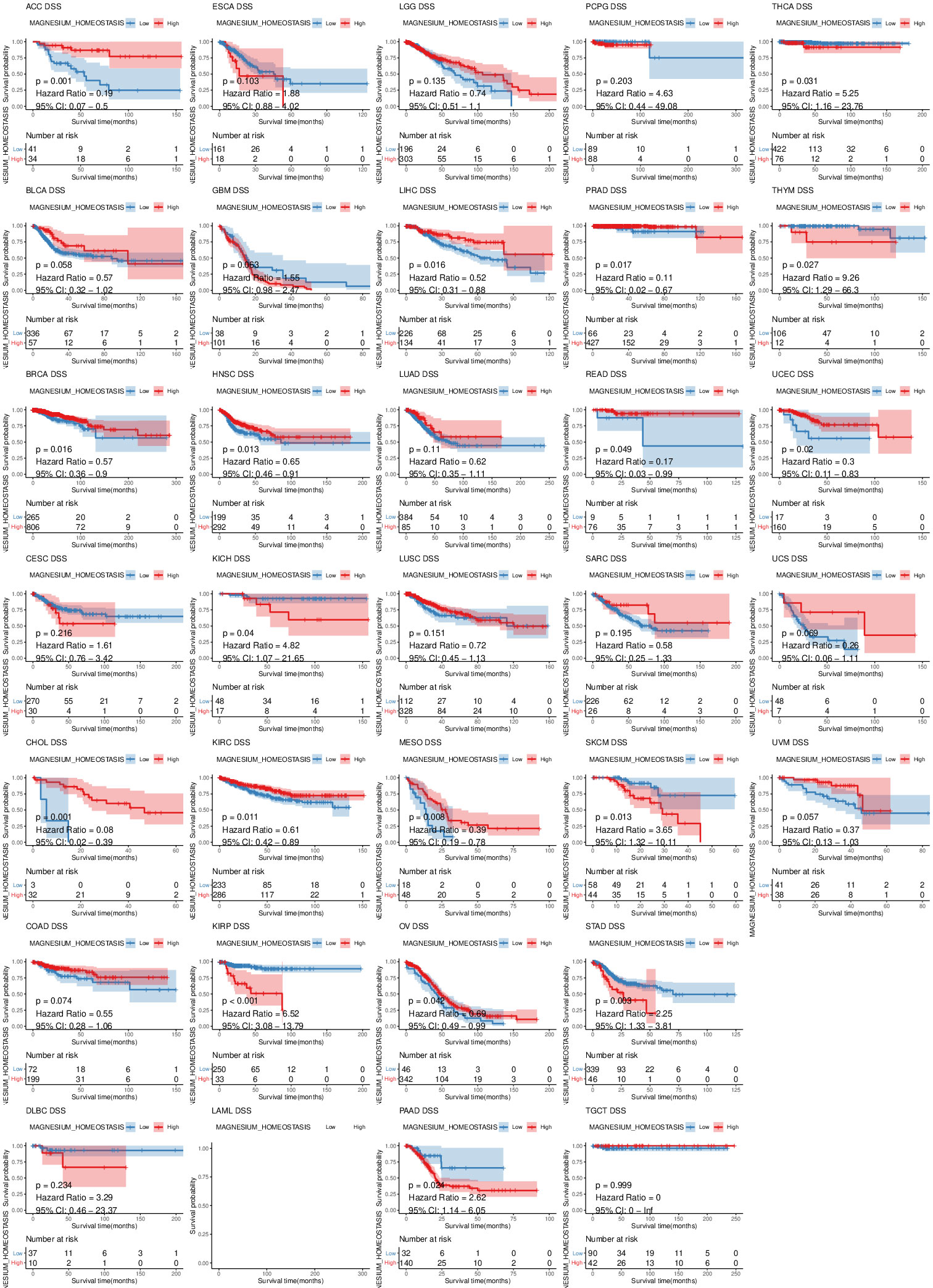
**

**Supplementary Figure 4** Effect of magnesium homeostasis on overalldisease-specific survival (OSDSS).

**
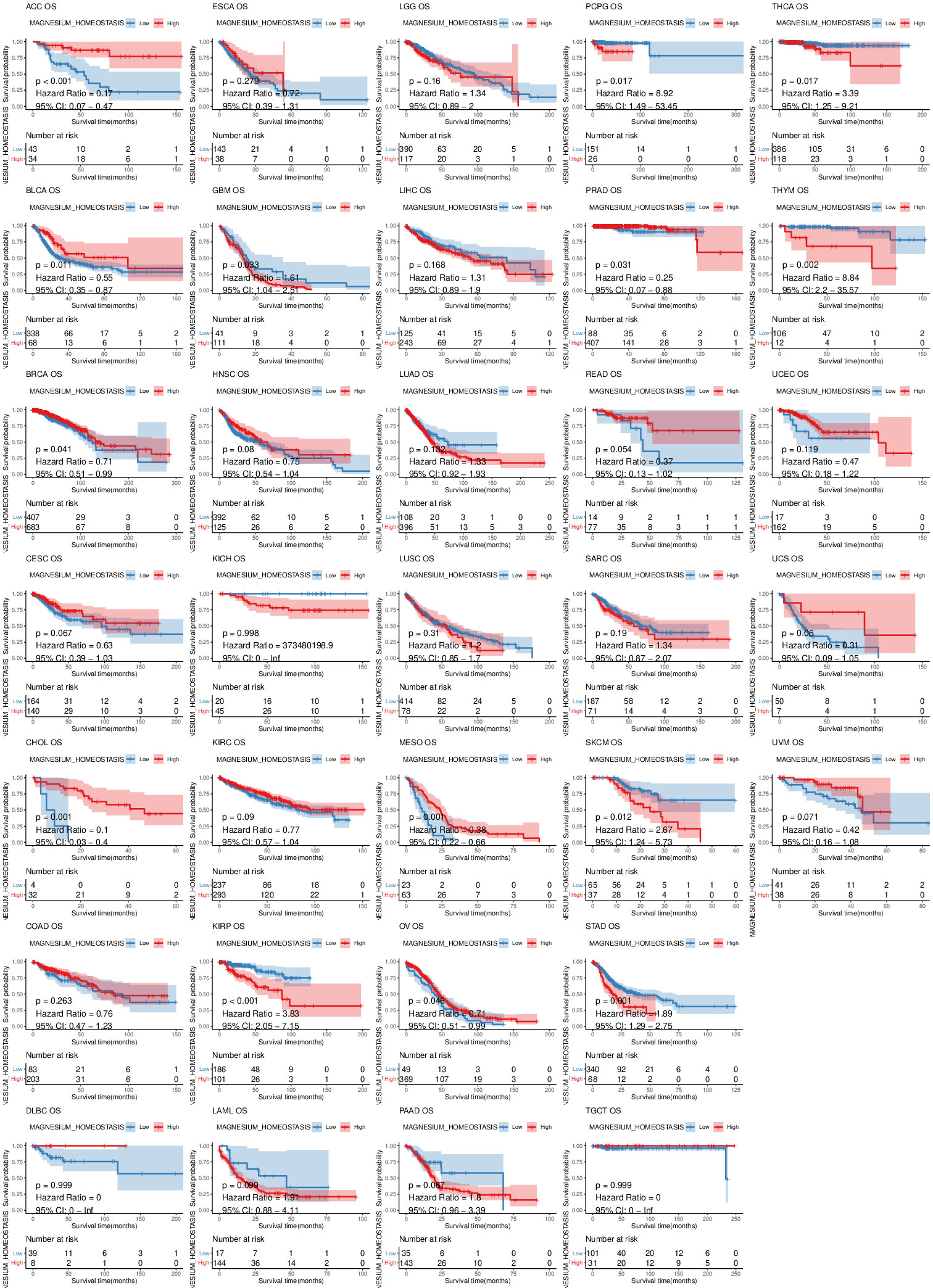
**

**Supplementary Figure 5** Effect of magnesium homeostasis on disease-free interval (DFI).

**
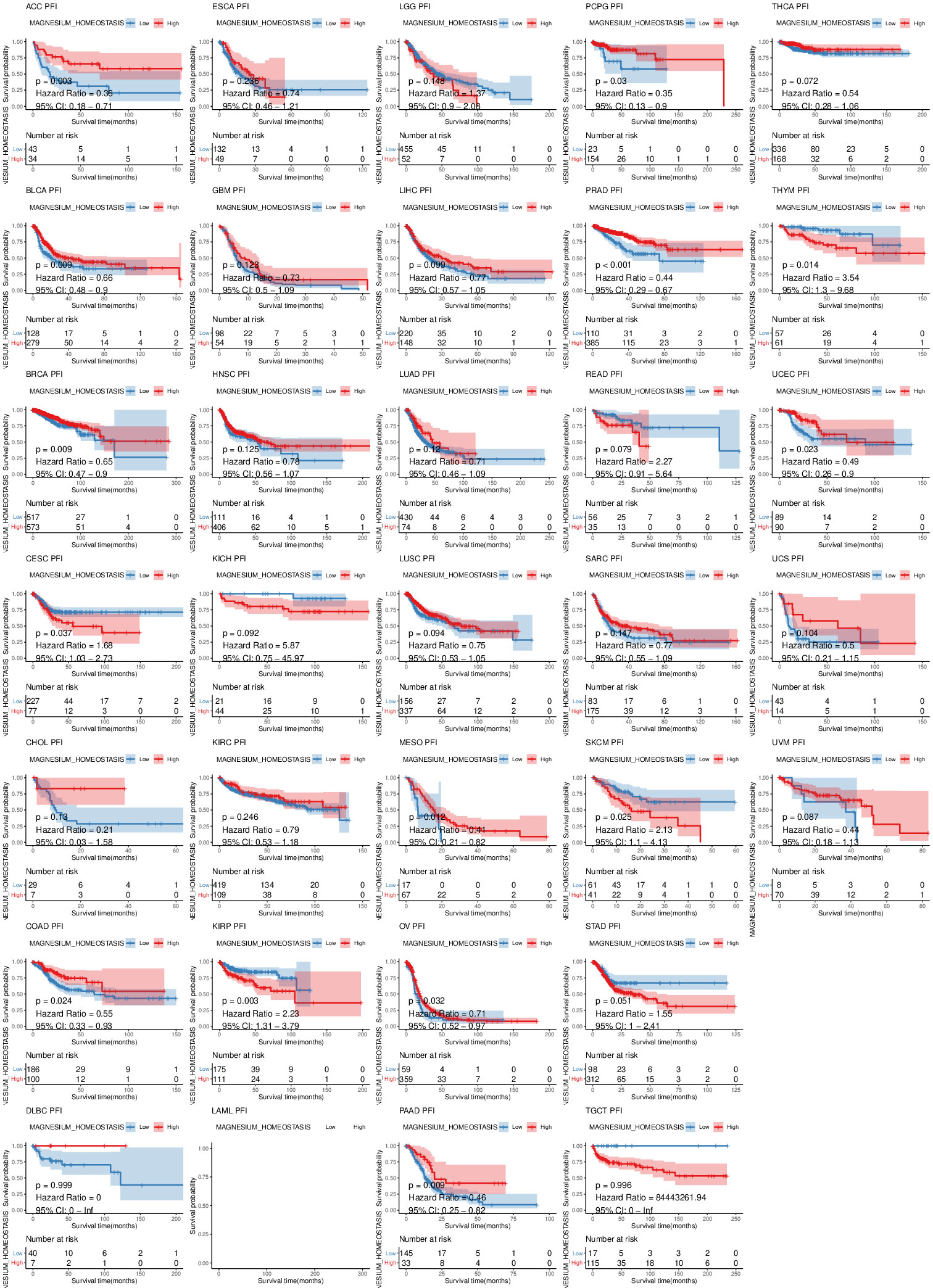
**

**Supplementary Figure 6** The relationship between magnesium homeostasis scoresscore and cancer stromal score.

**
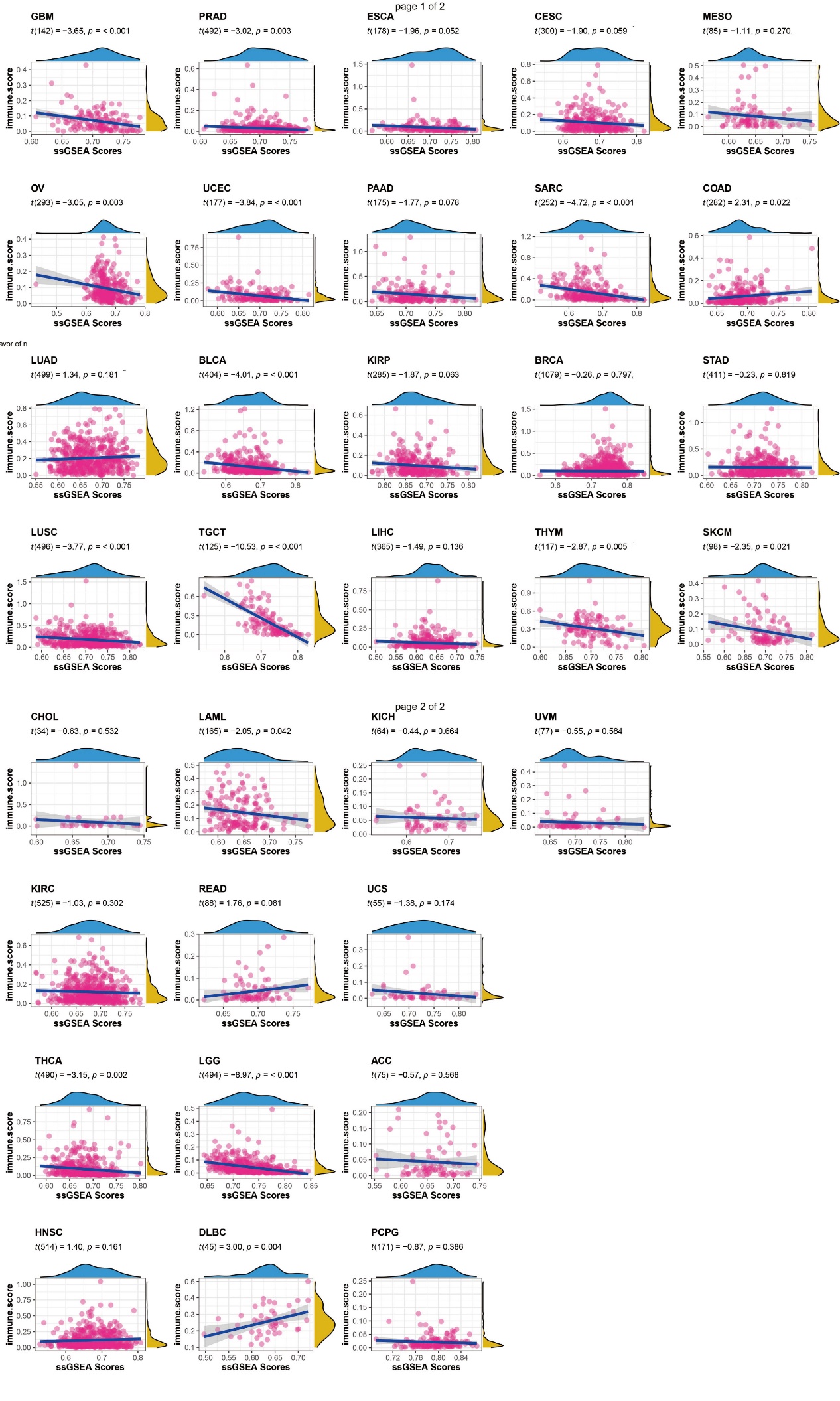
**

**Supplementary Figure 7** The relationship between magnesium homeostasis scoresscore and cancer immune score.

**
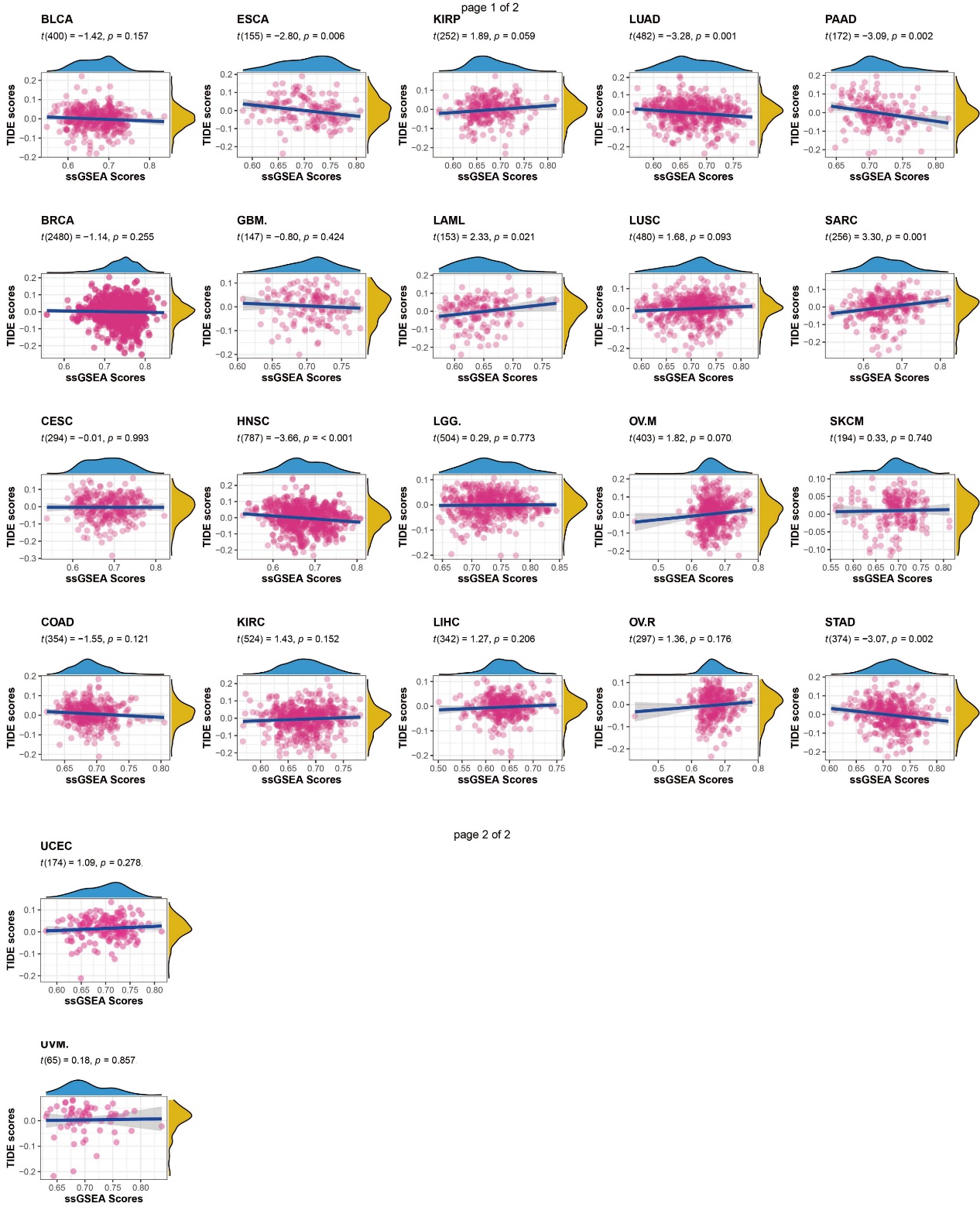
**

**Supplementary Figure 8** The relationship between magnesium homeostasis scoresscore and cancers microenvironment score.

**
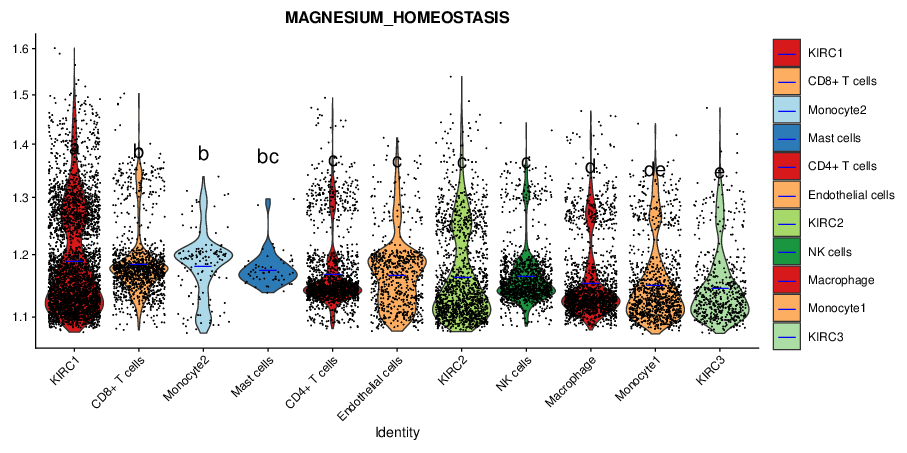
**

**Supplementary Figure 9** Expression level of different markers in different cells.

**
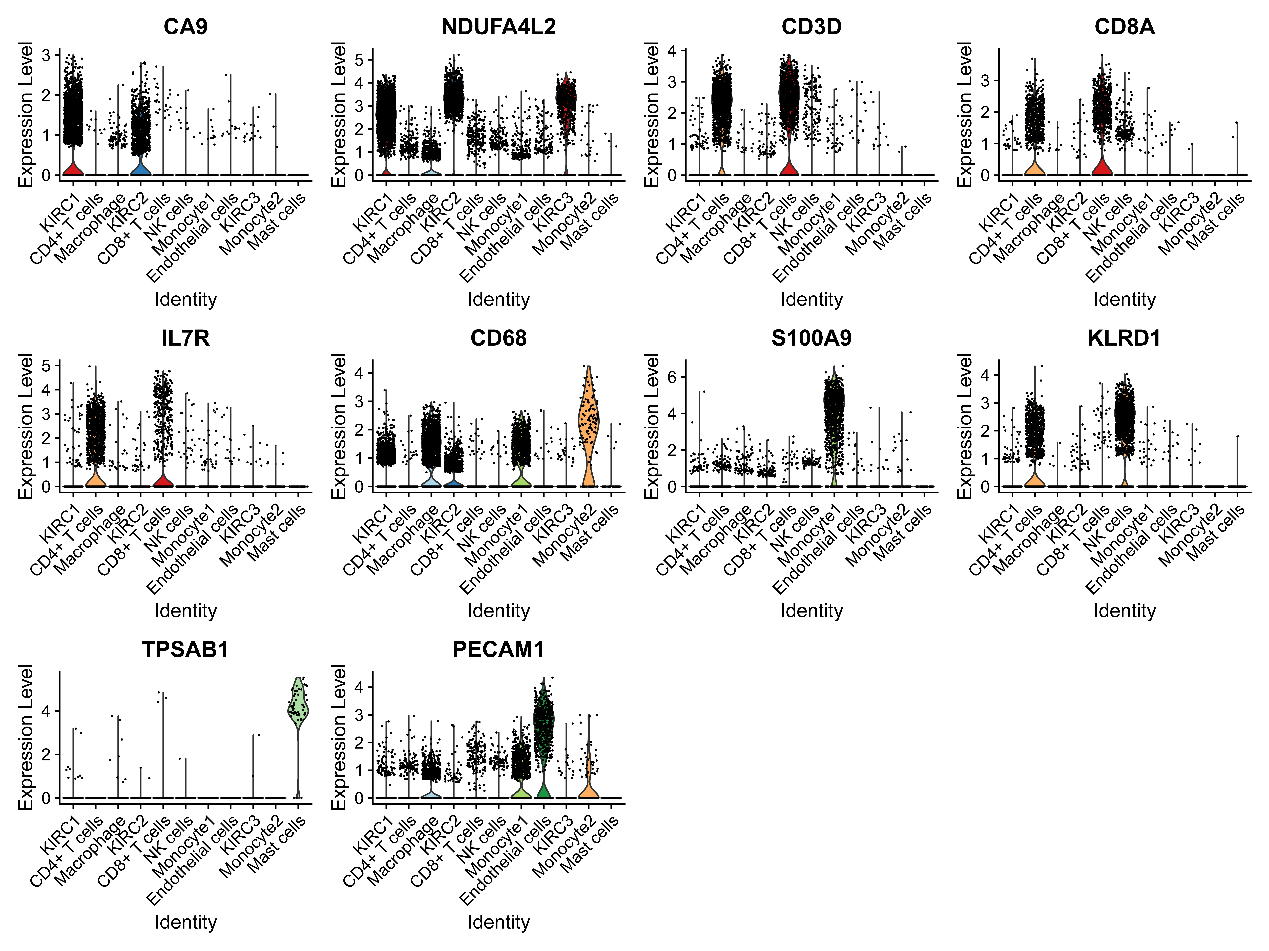
**

**Supplementary Figure 10** The marker genes expressed in different cell populations.


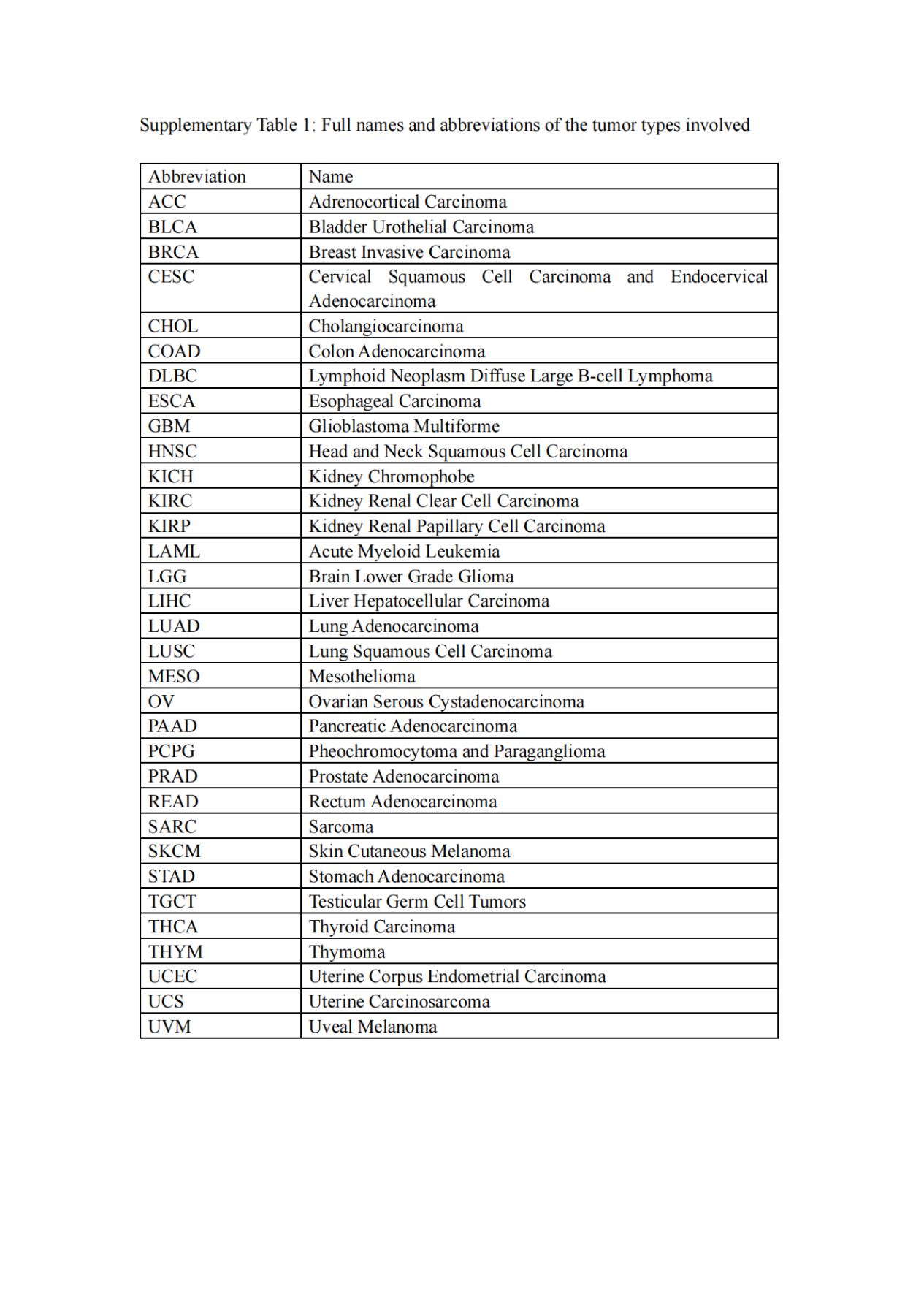


Supplementary Table 1: Full names and abbreviations of the tumor types involved
